# CAR Gene Delivery by T‐cell Targeted Lentiviral Vectors is Enhanced by Rapamycin Induced Reduction of Antiviral Mechanisms

**DOI:** 10.1002/advs.202302992

**Published:** 2023-10-30

**Authors:** Filippos T Charitidis, Elham Adabi, Naphang Ho, Angela H Braun, Ciara Tierney, Lisa Strasser, Frederic B Thalheimer, Liam Childs, Jonathan Bones, Colin Clarke, Christian J Buchholz

**Affiliations:** ^1^ Molecular Biotechnology and Gene Therapy Paul‐Ehrlich‐Institut 63225 Langen Germany; ^2^ Deutsches Krebsforschungszentrum and German Cancer Consortium (DKTK) 69120 Heidelberg Germany; ^3^ Characterisation and Comparability Laboratory National Institute for Bioprocessing Research and Training Foster Avenue, Mount Merrion, Blackrock Dublin A94 X099 Ireland; ^4^ Frankfurt Cancer Institute (FCI) Goethe University 60590 Frankfurt am Main Germany; ^5^ Host‐Pathogen Interactions Paul‐Ehrlich‐Institut 63225 Langen Germany; ^6^ School of Chemical and Bioprocess Engineering University College Dublin D04 V1W8 Belfield Dublin Ireland; ^7^ National Institute for Bioprocessing Research and Training A94×099 Foster Avenue, Mount Merrion, Blackrock Dublin Ireland

**Keywords:** CAR T cells, IFITM, *in vivo* gene delivery, rapamycin, receptor‐targeted vectors, transduction enhancer, scRNA‐seq

## Abstract

Lentiviral vectors (LV) have become the dominant tool for stable gene transfer into lymphocytes including chimeric antigen receptor (CAR) gene delivery to T cells, a major breakthrough in cancer therapy. Yet, room for improvement remains, especially for the latest LV generations delivering genes selectively into T cell subtypes, a key requirement for in vivo CAR T cell generation. Toward improving gene transfer rates with these vectors, whole transcriptome analyses on human T lymphocytes are conducted after exposure to CAR‐encoding conventional vectors (VSV‐LV) and vectors targeted to CD8+ (CD8‐LV) or CD4+ T cells (CD4‐LV). Genes related to quiescence and antiviral restriction are found to be upregulated in CAR‐negative cells exposed to all types of LVs. Down‐modulation of various antiviral restriction factors, including the interferon‐induced transmembrane proteins (IFITMs) is achieved with rapamycin as verified by mass spectrometry (LC‐MS). Strikingly, rapamycin enhances transduction by up to 7‐fold for CD8‐LV and CD4‐LV without compromising CAR T cell activities but does not improve VSV‐LV. When administered to humanized mice, CD8‐LV results in higher rates of green fluorescent protein (GFP) gene delivery. Also in vivo CAR T cell generation is improved in kinetics and tumor control, however to a moderate extent, leaving room for improvement by optimizing the rapamycin administration schedule. The data favor multi‐omics approaches for improvements in gene delivery.

## Graphical abstract

The work highlights the benefit of single‐cell‐based monitoring approaches for the identification of restriction factors inhibiting lentiviral‐vector‐mediated transduction. By downmodulating those factors both, conventional and in vivo CAR T cell generation by T‐cell targeted vectors can be improved.

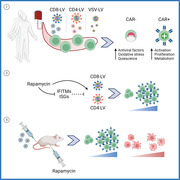

## 1 Introduction

Gene therapy aims at transferring exogenous genetic material to a therapeutically relevant cell of interest to recover a compromised biological function or to introduce a completely new protein, such as the chimeric antigen receptor (CAR).^[^ ^1^ ^]^ Delivery of CARs into patients' T lymphocytes often relies on retro‐ or lentiviral vectors (LVs). Primary T lymphocytes are highly heterogeneous, e.g., with respect to their phenotype or activation status. Accordingly, gene transfer rates vary significantly. Usually, less than half of the T cells convert into CAR T cells after exposure to vector particles. In the authorized product Yescarta for instance, only an average of 37% of the T cells are CAR‐positive (EMA/481168/2018).^[^ ^2^ ^]^ Due to a complicated manufacturing process resulting in an individualized autologous medicinal product, the in vivo generation of CAR T cells is currently moving into focus.^[^ ^3^ ^]^ For both, ex vivo and in vivo gene delivery, the improvement of gene transfer rates into primary T lymphocytes without compromising safety and cost‐effective production holds considerable promise for the gene therapy field.

LVs pseudotyped with the vesicular stomatitis virus (VSV) glycoprotein (G) have become the most common and conventional way of transducing human T cells. The entry receptor of VSV, the low‐density lipoprotein receptor (LDLR), is expressed in all human cell types with the exception of unstimulated human T lymphocytes.^[^ ^4^, ^5^ ^]^ This together with its broad tropism prevents the use of VSV‐LV for in vivo CAR administration and has driven the development of engineered envelopes enabling LVs to use T cell surface markers as entry receptors.^[^ ^5^, ^6^ ^]^ For this technology, paramyxoviral attachment proteins, such as the Nipah virus protein G or the measles virus hemagglutinin are blinded for natural receptor recognition and conjugated with a single‐chain variant (scFv) or a designed ankyrin repeat protein (DARPin) providing high‐affinity target receptor binding. The respective fusion protein mediates membrane fusion and release of the pre‐integration complex into the cell. Among the recently developed vectors are LVs using human CD8 or CD4 as entry receptors.^[^ ^7^, ^8^, ^9^ ^]^ The corresponding CD8‐LV and CD4‐LV achieve highly selective gene transfer into their respective T lymphocyte subtypes with more than 99% target cell specificity.^[^ ^10^, ^11^ ^]^ Notably, when being administered separately or combined for in vivo CD19‐CAR T cell generation, a single injection was sufficient to eradicate CD19‐positive tumors in NSG humanized mice.^[^ ^12^ ^]^

Conventional and receptor‐targeted LV types, rely on membrane fusion to enter the cells and integrate the transferred genetic information. VSV‐LV hijacks the pH‐dependent endosomal pathway. Upon clathrin‐mediated endocytosis, pH reduction in the endosomes activates VSV‐G, which penetrates the membrane for the release of the genetic material into the cell.^[^ ^13^, ^14^, ^15^ ^]^ Unlike VSV‐LV, LVs pseudotyped with paramyxoviral envelope proteins, such as CD4‐LV and CD8‐LV, fuse directly with the cell membrane and release their genetic material in this pH‐independent way.^[^ ^16^ ^]^ Due to their human immunodeficiency virus (HIV)‐derived origin, all types of LVs need to bypass cell‐intrinsic barriers that can affect their route to integration and transgene expression. Steps such as viral entry, reverse transcription, nuclear transport, integration, and transgene expression can be impacted by induced antiviral defense mechanisms including cellular restriction factors.^[^ ^17^, ^18^ ^]^

Alleviating such intrinsic antiviral restriction mechanisms with small compounds can enhance the infectivity of viruses, including LV‐mediated gene transfer. Such an example is the immunosuppressive anti‐cancer drug rapamycin. On hematopoietic stem and progenitor cells (HSPC) rapamycin has been shown to enhance genetic modification by VSV‐LV through downmodulation of interferon‐induced restriction factors.^[^ ^19^, ^20^ ^]^ Its benefit for the transduction of other cells including human T lymphocytes has not been investigated.

Recently, we have established a single‐cell transcriptomics approach to monitor the conversion of T cells into CAR T cells upon transduction by LVs. Restricted to a panel of 399 immuno‐oncology‐related genes plus the CAR mRNA derived from the integrated LV, target cell specificity of more than 99% was determined for CD8‐LV. Using this method, we observed the upregulation of genes including those encoding restriction factors as potentially causative for the failure of proper LV‐mediated transduction in CAR‐negative cells.^[^ ^11^ ^]^ Among them were IFITM2 and IFITM3, which are present in endosomal compartments and known to inhibit membrane fusion of the viral envelope with endosomal membranes.^[^ ^21^, ^22^ ^]^

In this study, we conducted whole transcriptome analysis on human T lymphocytes early after exposure to CAR‐encoding VSV‐LV, CD8‐LV, or CD4‐LV. Genes encoding antiviral restriction factors were found to be upregulated in CAR‐negative cells and treatment of T lymphocytes with the immunosuppressant agent rapamycin, which resulted in downmodulation of various antiviral restriction factors including IFITMs as determined by proteome analysis. While rapamycin did not improve transduction with VSV‐LV, it resulted in substantial enhancement of transduction efficiencies achieved with CD8‐LV and CD4‐LV. Administration of rapamycin to PBMC‐humanized mice prior to CD8‐LV injection increased in vivo reporter as well as CAR gene transfer rates considerably. The latter resulted in superior in vivo CAR T cell generation and tumor remission.

## 2 Results

### 2.1 Distinct Gene Expression Profiles in CAR T Cells

**Figure 1:**
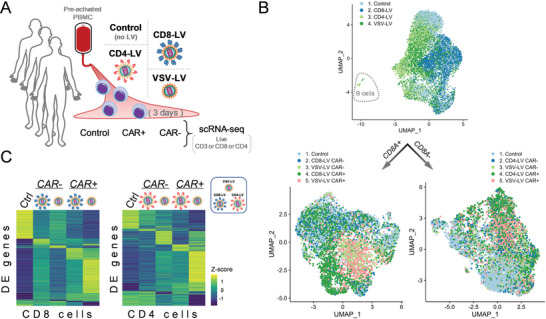
Distinct gene expression profiles in T cells exposed to different vector types. A) Experimental layout of scRNA‐seq analysis. Pre‐activated PBMC isolated from three donors were incubated with VSV‐LV, CD8‐LV, or CD4‐LV and cultivated for 3 days before sorting and processing for WTA. Cultured T cells that did not come across with any LV were used as a control. B) UMAP plots of all processed cells from the four conditions (*n =* 13178), which were computationally further separated into CD8 and CD4 cells for downstream analysis (*n =* 5777 and *n =* 6848, respectively). C) Heatmap plots of all differentially expressed genes (DEGs) per cluster identified by the Seurat function FindAllMarkers, in CD8 (left) and CD4 (right) cell populations (Wilcoxon sum rank test, |log_2_FC| > 0.2, FDR < 0.05). *z*‐scores were calculated from averaged gene expression per cluster.

For the whole transcriptome (WTA) approach on a single‐cell level we incubated pre‐activated PBMC from 3 donors with CD8‐LV, CD4‐LV, or VSV‐LV encoding CD19‐CAR‐28z. Cultured T cells that did not come across with any LV were used as a control (**Figure** **1**A). In total, 15334 cells were analyzed by sequencing with, on average, 29229 reads per cell. Since target selectivity in this setting had been determined to be over 99% for CD8‐LV,^[^ ^11^ ^]^ we focused on the identification of potential blocks preventing successful CAR delivery to particular T cells, the cells were magnetically sorted 3 days after vector exposure into live CD8 cells (CD8‐LV), CD4 cells (CD4‐LV) and CD3 cells (VSV‐LV, control) and then processed for single‐cell RNA sequencing (scRNA‐seq) (Figure 1A).

UMAP plots of all three donors in all experimental settings revealed a nearly even distribution of the VSV‐LV sample, while the magnetically sorted CD8‐LV and CD4‐LV exposed samples separated into the CD8 and CD4 populations, as expected (Figure 1B, Figure S1, Supporting Information). Based on the expression thresholds obtained from the multimodal analysis, samples were subsetted into CAR‐positive and CAR‐negative CD8 or CD4 cells (Figure 1B, Figure S2, Supporting Information). Some remnant B cells clustering separately and identified by *CD19*, *MS4A1*, *CD22*, and *BASP1* expression were excluded from the downstream subsetting and analyses (Figure 1B). Interestingly, CD8 cell subsets exposed to the same type of LV clustered together, rather than T cells having the same transduction status (*CAR^−^* or *CAR^+^*) but originating from different LVs (Figure 1B, Figure S3A, Supporting Information). This was different for the CD4 cells, where both LV‐inoculated samples overlaid almost evenly (Figure 1B, Figure S3B, Supporting Information). In addition, while control CD8 cells did not form specific clusters and roughly overlaid the CD8‐LV cell populations, control CD4 cells showed clusters distinct from any other cell population as a result of abundant transcriptional diversity (Figure 1B, Figure S3B,C, Supporting Information). Differential gene expression analysis revealed transcriptomic alterations between control cells and cells exposed to LVs as well as between *CAR^+^* and *CAR^−^* cells (Figure 1C). Furthermore, each particular type of LV caused distinct transcriptomic patterns either in *CAR^+^* or *CAR^−^* cells. *CAR^+^* cells generated by VSV‐LV showed an induction of a greater number of genes when compared to the average expression of all other analyzed subsets (Figure 1C). When exploring the differences between control cells versus the bulk LV‐inoculated samples, CD8‐LV had the least effect on transcriptional changes, altering the expression of 213 genes, whereas both, VSV‐LV and CD4‐LV, affected at least two times more genes (Figure S4A, Supporting Information). Examining the common 83 differentially expressed genes (DEG) altered upon LV inoculation, they were associated with metabolic pathways, cell proliferation, and viral infection (Figure S4B, Supporting Information).

**Figure 2:**
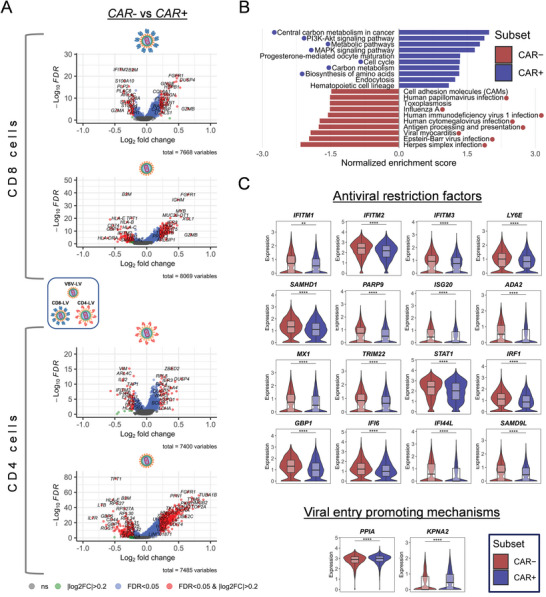
Transcriptomic differences between *CAR^−^* and *CAR^+^* cells. A) Volcano plots of DEGs between *CAR^+^* and *CAR^−^* cells in CD8‐LV, CD4‐LV, or VSV‐LV exposed CD8+ and CD4+ cell populations, respectively (Wilcoxon rank sum test). Negative fold‐change indicates upregulation in *CAR* ^−^ cells and vice versa. (Bullets; black: non‐significant, green: |log_2_FC| > 0.2, blue: FDR < 0.05, red: |log_2_FC| > 0.2 & FDR < 0.05). FDR: false discovery rate. B) Gene set enrichment analysis (GSEA) on Kyoto Encyclopedia of Genes and Genomes (KEGG) pathways of the DEGs when comparing *CAR^+^* and *CAR^−^* cells of all LV inoculated samples, disregarding T cell type (|log_2_FC| > 0.2, FDR < 0.05). Blue and red bullets indicate the biologically relevant pathways identified to be associated with the upregulated genes in either *CAR^−^* (red) or *CAR^+^* (blue) cells. C) Violin plots of differentially expressed genes of interest plotted for concatenated *CAR^−^* (red) and *CAR^+^* (blue) cell populations from all samples. Statistics show the FDR corrected values of differential expression analysis (Wilcoxon rank sum test). FDR: false discovery rate, **p<0.01, ***p<0.001, ****p<0.0001.

Focusing on the gene expression profiles regulating the susceptibility of T cells for LV‐mediated transduction, differential gene expression analyses were performed between *CAR* ^+^ and *CAR* ^−^ cells from each particular LV product and T cell type (**Figure** **2**A). The shared number of expressed genes among the individual analyses is plotted in Figure S4A (Supporting Information). Similar to what was observed in heatmap plots, VSV‐LV induced greater transcriptional changes than the two targeted LVs, when comparing *CAR^−^* versus *CAR^+^* cells (Figure S5A, Supporting Information, Figure 2A).

Gene set enrichment analysis of DEGs comparing the total *CAR^+^* and *CAR^−^* cells was performed thereby disregarding the T cell subtypes and the LVs used (Figure S5B, Supporting Information; Figure 2B). Genes upregulated in *CAR^+^* cells matched with pathways related to metabolism, cell cycle progression, amino acids biosynthesis, and signaling, which is in agreement with an activated cellular state and proliferation, which is expected from CAR‐mediated activities (Figure 2B). On the other hand, DEGs in *CAR^−^* cells related to viral infections, antigen presentation, and interferon signaling indicate a prominent antiviral defense status present in cells not successfully transduced by the LVs (Figure 2B). Overall, similar pathways were found to be associated with the differentially expressed genes between *CAR^−^* and *CAR^+^* cells of each LV sample and T cell type (Figure S6, Supporting Information). Thus, these GSEA analyses indicated that common genes and pathways are regulated leading to successful transduction of PBMC or inhibition of gene transfer, disregarding the vector type used and cell type targeted. Even though 23 genes were found to be shared between all the possible comparisons of *CAR^+^* versus *CAR^−^* cells (Figure S5A, Supporting Information), we identified significant DEGs exhibiting antiviral activity or even promoting viral infectivity (Figure 2C).

A significant number of interferon‐stimulated genes (ISGs) were found to be upregulated in *CAR^−^* cells, known to establish antiviral activity against various RNA and DNA viruses with a special focus on HIV, severe acute respiratory syndrome coronavirus 2 (SARS‐CoV‐2), measles (MV), influenza A, hepatitis C (HCV) and poxviruses. Notably, *IFITM1*, *IFITM2*, *IFITM3*, and *LY6E*, all inhibiting viral entry, were significantly increased in *CAR^−^* cells (Figure 2C). In addition, genes restricting reverse transcription (*SAMHD1*), discriminating and digesting non‐self RNA or DNA (*PARP9*, *ISG20*, *ADA2*) were up in *CAR^−^* cells (Figure 2C). Antiviral transcriptional suppressors (MX1, TRIM22) were also found to be elevated in *CAR^−^* cells (Figure 2C). Moreover, transcriptional factors regulating IFN type I mediated antiviral protection (*STAT1*, *IRF1*) as well as other antiviral‐related factors (*GBP1*, *IFI6*, *IFI44L*, *SAMD9L*) were increased in *CAR^−^* cells (Figure 2C). On the other hand, cyclophilin A (*PPIA*) and karyopherin subunit alpha 2 (*KPNA2*), which fulfill a crucial role in capsid trafficking and nuclear entry of the HIV pre‐integration complex (PIC), were found to be slightly upregulated in *CAR^+^* cells (Figure 2C).^[^ ^23^, ^24^ ^]^ Interestingly, all LVs induced genes are associated with DNA repair, the p53 pathway, unfolded protein response, reactive oxygen species, and apoptosis. These potential biomarkers were to a lower extent upregulated with the receptor‐targeted LVs than with VSV‐LV (Figure S8, Supporting Information). A detailed list of all genes grouped into thematic biological functions and their expression across each specific analyzed subset of cells is presented in Figure S8 (Supporting Information).

### 2.2 Rapamycin Enhances Transduction of CD8‐LV and CD4‐LV

**Figure 3:**
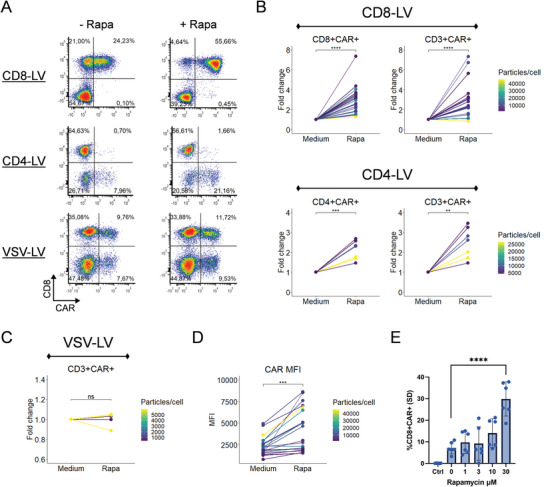
*Ex vivo* transduction enhancement by rapamycin. A) Representative flow cytometry results of CAR T cells generated with CD8‐LV, CD4‐LV, or VSV‐LV in presence or absence of 30 µM rapamycin. B) Fold change differences in the percentages of CAR^+^ cells upon transduction of the targeted T cell subtype (left) or total CD3 cells (right) with CD8‐LV (*n =* 16‐18, donors = 4) or CD4‐LV (*n =* 7, donors = 2) in presence or absence of 30 µM rapamycin. C) Effect of rapamycin on transducing CD3 cells with VSV‐LV (*n =* 5, donors = 2). D) Paired‐wise comparison of mean fluorescence intensity (MFI) of CAR expression in CAR T cells generated by CD8‐LV with or without 30 µM rapamycin (*n =* 18, donors = 4). E) Titration of rapamycin while using same CD8‐LV dose. Control cells were not inoculated (*n =* 6 per condition, donors = 2). Multiple comparisons were performed with ANOVA statistical tests between 0 µM and all the other conditions. ns: non‐significant, **p<0.01, ***p<0.001, ****p<0.0001.

To determine if the antiviral status in T cells could be altered, specifically the reduction of IFITM proteins, we added rapamycin during the incubation of human T cells with the different vector types. We observed a strong increase in gene delivery and thus in conversion of T cells into CAR T cells with both, CD8‐LV and CD4‐LV (**Figure** **3**A,B). The increase accounted for up to 7.3‐fold for CD8‐LV and 3.5‐fold for CD4‐LV and was more pronounced for lower particle doses, reaching transduction rates of more than 90% of the CD8 target cells or more than 50% of CD4 target cells, respectively (Figure 3A,B). Notably, rapamycin did not increase transduction mediated by VSV‐LV (Figure 3A,C).

Focusing more on the effects of rapamycin on CD8‐LV, we observed a significantly higher mean fluorescence intensity (MFI) of extracellular CAR, showing that rapamycin improved the expression of CAR on the cell surface (Figure 3D). Furthermore, 30 µM rapamycin was identified as optimal, while not impairing cell viability (Figure 3E; Figure S9, Supporting Information) or selectivity for target cells (Figure S10, Supporting Information).

### 2.3 Rapamycin Downmodulates Various Antiviral Restriction Factors in Human T Cells

**Figure 4:**
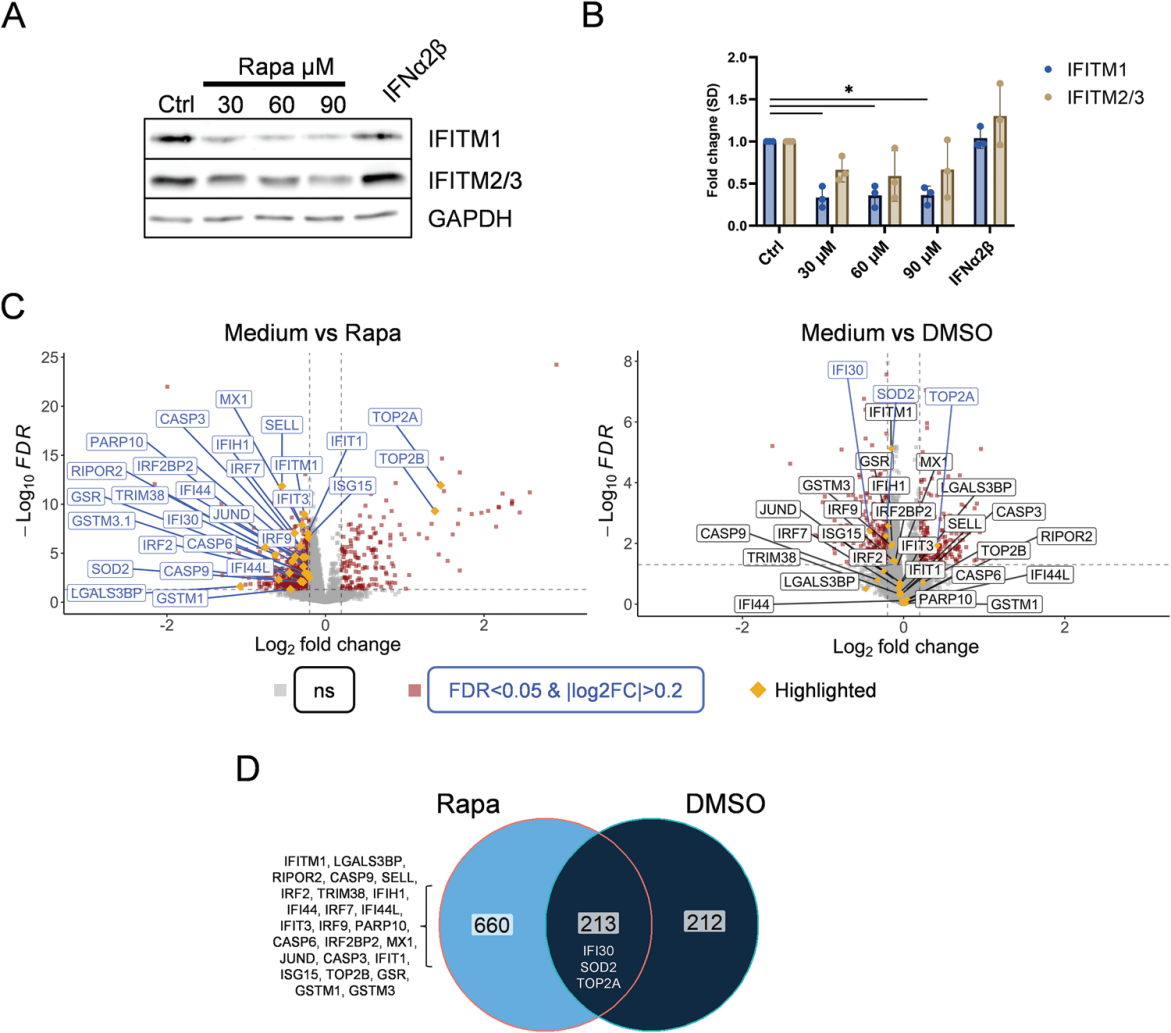
Proteomic alterations induced in human T cells by rapamycin. A) Western blot detecting IFITM1, IFITM2/3, and GAPDH in lysates of human T cells treated with increasing concentrations of rapamycin or 500 IU mL^−1^ IFNα2β for 1.5 h. B) Quantification of three western blots showing fold change differences of IFITMs normalized to GAPDH (donors = 3). Statistical analysis was performed by a two‐way ANOVA test. C) Volcano plots of LC‐MS proteome analysis of PBMC treated with 30 µM rapamycin versus untreated (donors = 6, in technical triplicates) (left), and, PBMC treated with 0.5% DMSO versus untreated (donors = 6, technical replicates of each sample = 3) (right). Red squares and dark blue text boxes indicate the significantly differentially regulated proteins (|log_2_FC| > 0.2, FDR < 0.05). Among them, proteins related to apoptosis, T cell quiescence, proliferation, oxidative stress, and IFN‐mediated antiviral inhibition are framed and highlighted by dark golden diamonds. Grey squares and black text boxes label proteins that are non‐significantly up‐ or downregulated. D) Overlap of significantly up‐ and downregulated proteins between rapamycin or DMSO treated samples (|log_2_FC| > 0.2, FDR < 0.05). *p<0.05.

To confirm the activity of rapamycin on downregulating IFITMs in human T cells, we incubated PBMC of 3 donors with different concentrations and determined the cellular levels of all three IFITMs. While incubation with IFNα further stimulated their expression, all applied doses of rapamycin led to a strong downmodulation of all three IFITMs within 1.5 h (**Figure** **4**A,B). To further explore the consequences of rapamycin treatment in T lymphocytes on protein level, we conducted liquid chromatography‐mass spectrometry (LC‐MS) proteome analysis of pre‐activated PBMC from 6 donors, comparing untreated samples with rapamycin or DMSO (diluent) treated samples, respectively. To our surprise, not only IFITM1 protein was downregulated upon rapamycin treatment thus confirming the western blot data, but 16 other antiviral proteins featuring the IFN type I antiviral pathway were decreased, too (Figure 4C). These included LGALS3BP, IFI30, IRF2, TRIM38, TRIM32, IFIH1, IFI44, IRF7, IFI44L, IFIT3, IRF9, PARP10, IRF2BP2, MX1, IFIT1 and ISG15. In addition, proteins related to a naïve (SELL) and quiescence (RIPOR2) cell state and T helper 2 (Th2) phenotype (JUND) were downregulated upon rapamycin treatment (see Supporting Information data set for detailed list), indicating either activation/mobilization or a selective pressure against naïve cells skewing the differentiation toward the Th1 phenotype.^[^ ^25^ ^]^ Also, some caspases were found to be downregulated (CASP3, CASP6, CASP9), while proliferation and DNA re‐organization related proteins (TOP2A, TOP2B) were upregulated, showing a possible anti‐apoptotic effect by rapamycin (Figure 4C). The downregulation of SOD2, GSR, GSTM1, and GSTM3 by rapamycin indicates susceptibility to oxidative stress (Figure 4C). However, at least in part, this came from DMSO used as a diluent of rapamycin. While DMSO did not significantly influence the antiviral proteins, with the exception of IFI30, it slightly contributed to the changes observed for SOD2 and TOP2A (Figure 4C). Overall, of the 882 proteins found to be dysregulated upon rapamycin treatment (711 down‐ and 171 upregulated) 213 out of 429 identified upon DMSO treatment were in common with the rapamycin‐treated samples, although the majority of differences was less significant and less pronounced in DMSO treated samples (Figure 4D).

### 2.4 Enhanced In Vivo GFP Transfer with CD8‐LV Upon Rapamycin Treatment

**Figure 5:**
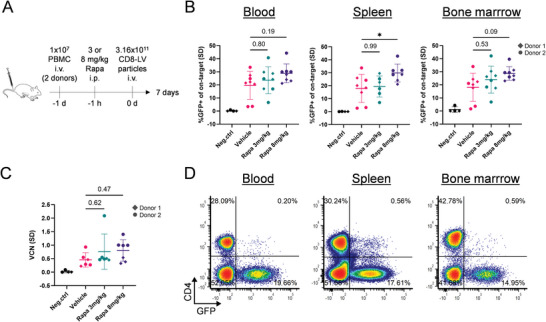
Enhancing in vivo GFP transfer with rapamycin. A) Experimental workflow of in vivo GFP transfer with CD8‐LV into humanized NSG mice. Rapamycin was injected in concentrations of 3 or 8 mg kg^−1^. Vehicle (2% DMSO, 30% PEG300, 5% Tween‐80) was used as control. The study was repeated with two anonymous donors. In vivo selectivity of CD8‐LV was determined on human CD3+/CD4‐ (on‐target) and human CD3+/CD4+ cells (off‐target). B) GFP expression on human CD8 on‐target cells assessed by flow cytometry in blood, spleen, and bone marrow (*n =* 4–8). P‐values from one‐way ANOVA with Tukey's multiple comparisons test are displayed on the graphs. C) Vector copy numbers (VCNs) of GFP gene integration into genomic DNA of human CD3 cells sorted from mouse spleens. Statistical analysis was performed by one‐way ANOVA with Tukey's multiple comparisons. D) Flow cytometry plots of GFP expression of human CD3+ cells in concatenated tissue‐specific samples from rapamycin‐treated mice. i.v. intravenous, i.p. intraperitoneal. *p<0.05.

Having documented the huge potency of utilizing rapamycin for enhancing gene transfer in vitro as well as the downmodulation of various antiviral restriction factors in human PBMC, we next assessed its effect on in vivo gene delivery with CD8‐LV. For that purpose, immunodeficient NSG mice were transplanted with pre‐activated PBMC (1 × 10^7^ per mouse) one day prior to LV administration (**Figure** **5**A). The next day, two doses of rapamycin, or vehicle were administered. One hour later we injected CD8‐LV particles and monitored mice for 7 days. GFP expression was assessed by flow cytometry in peripheral blood, spleen, and bone marrow.

There was no influence of rapamycin or vehicle on the reconstitution of human T cells in the mice. Particularly, the fractions of human CD8 T cells in blood, spleen, and bone marrow were highly homogenous and did not differ between the groups (Figure S11A, Supporting Information). Intriguingly, we observed the overall highest levels of GFP in mice that had received the high rapamycin dose (8 mg kg^−1^) (Figure 5B). This was most pronounced in the spleen where statistical significance was reached, while blood and bone marrow were close to significance. Also, at the low dose, a slight increase in the percentage of GFP‐positive CD8+ cells over the vehicle group was documented (Figure 5B). Remarkably, transduction rates in the high‐dose group were much more consistent than in the vehicle or low‐dose groups (Figure 5B). The vector copy numbers (VCNs) present in splenic T lymphocytes and the MFIs of GFP expression further confirmed the in vivo transduction enhancement mediated by rapamycin (Figure 5C; Figure S11B, Supporting Information). Moreover, rapamycin did not perturb the in vivo specificity of CD8‐LV for human CD8 cells, which reached 99.0% (±1.6%) in blood, 99.1% (±0.4%) in the spleen, and 98.9% (±0.8%) in bone marrow for rapamycin‐treated mice (Figure 5D, Table S1, Supporting Information). Notably, off‐target transduction of murine cells remained below detection level (Figure S12, Table S2, Supporting Information).

### 2.5 In Vivo CAR T Cell Generation with Rapamycin Shows Faster Tumor Regression

To apply our findings to a therapeutic approach, we next investigated if rapamycin improves CAR T cell generation with CD8‐LV. First, we assessed if rapamycin had any influence on CAR T cell properties beyond enhancing gene delivery. The presence of rapamycin did not result in any significant differences in proliferation rates (Figure S13A, Supporting Information). Also, their cytotoxicity against target Nalm‐6 cells was indistinguishable (Figure S13B, Supporting Information). Finally, the treatment did neither significantly impact the exhaustion of CAR T cells nor the memory phenotype mainly present in the majority of the analyzed donors (Figure S13C,D, Supporting Information).

**Figure 6:**
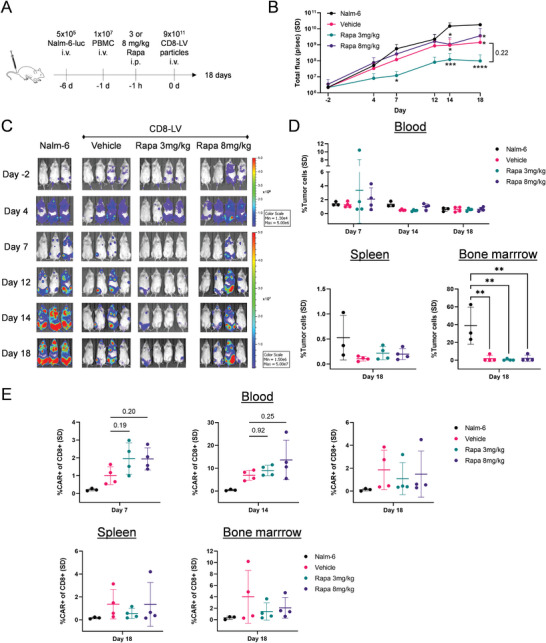
In vivo CAR T cell generation in presence of rapamycin. A) Experimental workflow. Rapamycin was injected in concentrations of 3 or 8 mg kg^−1^ and vehicle (2% DMSO, 30% PEG300, 5% Tween‐80) was used as control. As further control (Nalm‐6) animals received tumor cells but no vector. B) Tumor burden determined by luminescence. Mice were monitored at the indicated days pre‐ and post CD8‐LV and rapamycin injection. Two‐way ANOVA with Tukey's multiple comparisons was performed on log‐transformed data (*n =* 3‐4 per group). Numerical *p*‐value indicates the statistical result at the experimental endpoint. C) Luminescence imaging showing the ventral side of the mice. D) Frequencies of Nalm‐6 (CD19+) tumor cells in the indicated mouse tissues as detected by flow cytometry. One‐way ANOVA with Tukey's multiple comparisons was performed for statistical analysis. E) CAR expression out of human CD8 cells assessed by flow cytometry in blood and tissues at the indicated days. P‐values from one‐way ANOVA with Tukey's multiple comparisons are displayed on the graphs. i.v. intravenous, i.p. intraperitoneal. *p<0.05, **p<0.01, ***p<0.001, ****p<0.0001.

For the in vivo setting (**Figure** **6**A), mice were transplanted with Nalm‐6‐luc cells stably expressing firefly luciferase. After 4 days, mice were allocated to the four different groups resulting in a similar distribution of tumor load (Figure S14A, Supporting Information). Pre‐activated human PBMC were injected and the following day we administered vehicle or rapamycin in two different doses followed by i.v. injection of CD8‐LV transferring the CD19‐CAR‐28z gene. All vector‐treated groups reached tumor control with statistical significance by day 14 (Figure 6B,C). While the tumor in most of the treated mice progressed at rates comparable to the control until day 12 and then declined and reached a plateau, the low‐dose rapamycin mice were much more rapid in tumor control reaching this status already by day 7. In this group, tumor loads remained at substantially lower levels with higher statistical significance (*p<*0.0001–0.05) than the vehicle group (*p<*0.05) (Figure 6B). At the endpoint of the experiment, 2 out of 4 mice had no detectable luciferase signals in the 3 mg kg^−1^ group, while this was the case for 1 out of 4 mice in the two other vector‐treated groups (Figure 6C). One mouse of the 8 mg kg^−1^ group presented a higher tumor flux compared to all other LV‐treated mice. This was most likely due to having inadvertently injected more tumor cells, which is reflected by the highest tumor load at randomization (Figure S14A, Supporting Information). Yet, also this mouse did not reach the tumor levels of untreated mice, presenting at least some control of tumor growth (Figure 6B,C).

Flow cytometry was performed to monitor the cellular compositions in blood during intermediate samplings and additionally in spleen and bone marrow upon termination. Rapamycin had no impact on the frequencies of human CD45 and CD8 cells (Figure S14B,C, Supporting Information). As expected for this tumor model, a vast number of Nalm‐6 cells were present in bone marrow making up around 40% of all cells on average (Figure 6D). Only small amounts were detectable in the spleen and blood of the untreated control animals. In all vector‐treated animals, tumor cell levels were substantially reduced or even undetectable. The tumor cell elimination from bone marrow was clearly documented and was well in agreement with the data obtained by in vivo imaging. Residual tumor cells were only detectable in a single animal of the vehicle and the high‐dose group, respectively. On day 7 post vector injection CAR T cell levels in both rapamycin groups made up about 2% of CD8 cells, which was about two‐fold higher than in the vehicle group (Figure 6E), which nicely corresponded to the more rapid tumor cell clearance in the low dose group monitored by in vivo imaging. CAR T cell frequencies peaked on day 14 post‐injection in all vector groups. On day 18, CAR T cell levels had declined, thus correlating with tumor regression. In fact, it was the individuals with the highest remaining tumor loads that had detectable CAR T cells on that day, indicating a better expansion and persistence of CAR T cells in the presence of tumor target cells (Figure 6E). There was no off‐target CAR expression detectable neither in human nor murine cells derived from blood, spleen, or bone marrow (Figure S15, Supporting Information). Further proof for the successful in vivo generation of CAR T cells was provided by ex vivo co‐culturing mouse splenocytes and bone marrow cells harvested from the vector‐injected animals with irradiated tumor cells. CAR T cells quickly expanded in all groups with frequencies correlating well to those detected directly in the harvested organs (Figure S14D, Supporting Information). In addition, VCNs determined in human T lymphocytes on day 18 mirrored the unsynchronized decrease of CAR T cells between the LV groups, with the rapamycin‐treated mice having a faster decrease (Figure S14E, Supporting Information). Overall, rapamycin administration showed a potency to mediate CAR T cell generation with CD8‐LV more rapidly in vivo, controlling tumor growth quicker.

## 3 Discussion

In this study, we describe transcriptomic differences across the analyzed subsets of T cells inoculated with VSV‐LV, CD8‐LV, or CD4‐LV, transferring CD19‐CAR. We performed WTA of T cells especially early (72 h) after exposure to CAR‐encoding LVs. This time point was a compromise between covering as many as possible genes induced by vector‐particle contact on one hand and the accumulation of sufficient amounts of CAR mRNA in transduced cells to distinguish between CAR‐negative and CAR‐positive cells on the other hand. Beyond that, the use of different types of LVs facilitated the differentiation between genes induced by vector exposure from those induced by cultivation conditions or CAR activities. By focusing on the transcriptional differences between *CAR^−^* and *CAR^+^* cells, we identified common features in both, CD8 and CD4 T cells, associated with type I interferon‐induced pathways, further antiviral responses, and cell phenotypic and metabolic status (Figure 2B, Figure S6, Supporting Information). Among the genes found to be increased in *CAR^−^* cells, *IFITM1*, *‐2* and *‐3* are known to inhibit the fusion of enveloped viruses with cellular membranes, thus repressing their entry pathway (Figure 1E, Figure S7, Supporting Information).^[^ ^21^ ^]^ Therefore, we hypothesized that by downregulating the IFITMs, LV‐mediated transduction of T cells would be improved.

Inhibition of the mammalian target of rapamycin (mTOR) by its homonymous small molecule has been shown to decrease endosomal IFITM2 and IFITM3 expression, alleviating the antiviral restriction in human and mouse hematopoietic and progenitor stem cells (HSPC).^[^ ^19^, ^26^ ^]^ Even though rapamycin downregulated all three IFITMs in human T cells (Figure 4A,B), it did not increase VSV‐LV mediated transduction (Figure 3A,C). Intriguingly, rapamycin strongly improved gene transfer mediated by CD8‐LV and CD4‐LV, reaching up to 7.3‐fold and 3.5‐fold transduction enhancement, respectively (Figure 3A,B). Based on paramyxoviral glycoproteins, i.e., measles virus for CD4‐LV and Nipah virus for CD8‐LV, both these LVs fuse directly at the cell membrane,^[^ ^27^, ^28^, ^29^ ^]^ thus utilizing a different entry pathway than VSV‐LV. Several paramyxoviruses among them measles virus were previously shown to be inhibited by IFITM1, but not IFITM2 or IFITM3.^[^ ^30^ ^]^ The absence of activity by IFITM2 and IFITM3 was also demonstrated for the Nipah virus glycoproteins.^[^ ^31^ ^]^ While we do not provide direct evidence, the upregulation of IFITM1 in CAR‐negative cells, its downmodulation with rapamycin, and the plasma membrane entry pathway of the T‐cell targeted LVs make it likely that both were rather affected by IFITM1 than the other IFITMs. Independently from that, the enhanced gene delivery through rapamycin was likely the consequence of downmodulating an array of additional restriction factors.

Indeed, our proteome analysis of rapamycin‐treated T cells confirmed that multiple IFN‐induced antiviral restriction factors were downmodulated (Figure 4C). The anti‐apoptotic effect of rapamycin through mediating mitochondrial clearance by autophagy could be aligned with the reduction of caspases and an increase in topoisomerases.^[^ ^32^, ^33^ ^]^ The overall unchanged performance and minimally affected phenotype of CAR T cells generated in the presence of rapamycin demonstrates that this small molecule has the potential to be used as a transduction enhancer without negative consequences (Figure S13A–D, Supporting Information). Compared to its previous use on HSPC, we applied an approximately three‐fold higher dose with a substantially shortened exposure period. This is in line with a report on rapamycin‐treated HeLa cells, showing rapid reduction of IFITM3 levels starting at 30 min and reaching the maximum after 4 h.^[^ ^19^ ^]^

An important finding of our study with immediate implications for gene delivery is the enhancing effect of rapamycin without compromising the selectivity for in vivo gene delivery by CD8‐LV. Besides enhancement, the homogeneity of the reporter gene delivery rates in the presence of rapamycin was remarkable, while showing a huge spread in its absence in the vehicle control group. The data are especially encouraging since they were derived from pilot studies leaving ample room for optimization with respect to parameters such as route, dosage, and formulation. For instance, the presence of Tween 80 in the drug's vehicle formulation and its systemic bioavailability may have impacted the activity of LVs. Intraperitoneal injection was chosen as a way to extend the drug's half‐life keeping it bioavailable as long as possible. The lower dose of 3 mg kg^−1^ per mouse was extrapolated from the in vitro dose of 30 µM taking the mouse body surface into account.^[^ ^34^ ^]^ In addition, a higher dose of 8 mg kg^−1^ was used, which overall resulted in higher delivery rates most likely due to better bioavailability, which is compromised by rapamycin's high absorption to red blood cells.^[^ ^35^ ^]^ Both dosages were well tolerated with no adverse effects and continuous increase in weight (Figure S16, Supporting Information). The two applied dosages are equivalent to 8.9 mg m^−2^ and 24 mg m^−2^, respectively for 3 mg kg^−1^ and 8 mg kg^−1^, thus being well in the range of what is currently used in clinics (0.5–34 mg m^−2^). ^[^ ^34^ ^]^ However, to sustain the immunosuppressive or anticancer effects of rapamycin, the drug is usually administrated on a daily or weekly basis, while in our study we performed a single injection resulting in no signs of T cell depletion.

Also, for in vivo CAR delivery, rapamycin appeared to improve gene transfer and CAR T cell generation, thus accelerating tumor clearance. While the in vivo delivery of reporter genes provides a direct correlation between read‐out (GFP‐positive cells) and the enhancing effect of rapamycin, the situation is more complex with in vivo CAR delivery. The substantially higher number of transduced cells in blood upon GFP delivery cannot only be explained by the higher vector potency due to the smaller size of *GFP* compared to *CD19‐CAR*,^[^ ^36^ ^]^ CAR T cells migrate into tumor locations, in this case, the bone marrow, resulting in underestimated values of transduced cells in peripheral blood. Moreover, depending on the presence of target, i.e., tumor cells, CAR T cell peak numbers undergo a pronounced kinetic and do not run into a maximal steady‐state level as it is the case after reporter gene transfer. This explains why the enhancing activity of rapamycin was not reflected by CAR T cell numbers in lymphoid tissues harvested on the final day of the study. By then, CAR T cell numbers were already descending in the rapamycin groups but still high for the vehicle group. The reduction in tumor load was most likely solely due to the activity of the in vivo generated CAR T cells since there was no increased impact observed in the high dose group, while a slight reduction of the human T cell population was noticed on day 7 (Figure 6B,C). Notably, CAR T cells were successfully re‐isolated from mouse spleen and bone marrow of all vector‐receiving mice after ex vivo re‐stimulation (Figure S14D, Supporting Information). In agreement with the in vitro data, this argues for the rapamycin‐unaffected generation of memory CAR T cells in all groups.

Overall, incorporating cutting‐edge proteomic and single‐cell transcriptomic technology into CAR T cell therapy provides insights and essential aspects for product optimization in both conventional and targeted gene delivery. Transducing primary T cells, especially from cancer patients, remains to be challenging, frequently resulting in low‐efficiency rates.^[^ ^37^ ^]^ Thus, identification and downmodulation of restriction pathways have the potential to further improve the benefit provided by CAR T cell therapeutic strategies.

## 4 Experimental Section

### Cell Cultures

HEK‐293T (ATCC CRL‐11268) and HEK‐293T‐β2M^−/−^CD47^hi^ were cultured in Dulbecco's modified Eagle's medium (DMEM) (Sigma‐Aldrich, Munich, Germany) supplemented with 10% fetal calf serum (FCS) (Biochrom, Berlin, Germany) and 2 mM glutamine (Sigma‐Aldrich). MOLT‐4.8, A301, Nalm‐6 (ATCC CVCL_0092) and transgenic Nalm‐6 cells stably expressing firefly luciferase and enhanced blue fluorescent protein (eBFP) (Nalm‐6‐luc‐eBFP) were cultured in RPMI 1640 (Biowest, Nuaillé, France) supplemented with 10% FCS and 2 mM l‐glutamine. Cells were incubated at 37 °C with 5% CO_2_ and 90% humidity.

### Primary Cell Isolation, Culture, and Transduction

Blood units were purchased from the German Red Cross donation (DRK‐Blutspendedienst Baden‐Württemberg‐Hessen, Frankfurt) and used for the described work in agreement with the WMA Declaration of Helsinki and the Ethics Committee of the Goethe University Frankfurt (statement 20‐606). Human PBMC were isolated from peripheral blood buffy coats of four anonymous donors derived from density gradient centrifugation with Pancoll (PAN‐Biotech, Aidenbach, Germany), and purified PBMC were cryopreserved in 90% FCS and 10% dimethyl sulfoxide (DMSO, AppliChem GmbH, Darmstadt, Germany). PBMC were thawed and activated for 3 days in either T cell medium (RPMI 1640, 10% FCS, 2 mM l‐glutamine, 25 mM HEPES, 0.4% penicillin/streptomycin) or in 4Cell Nutri‐T medium (Sartorius AG, Gottingen, Germany) plus 0.4% penicillin/streptomycin with pre‐coated recombinant anti‐CD3 (1 µg mL^−1^, clone OKT3, Miltenyi Biotec, Bergisch Gladbach, Germany), soluble anti‐CD28 (3 µg mL^−1^, clone 15E8, Miltenyi Biotec), supplemented with the human cytokines IL‐7 (25 IU mL^−1^, Miltenyi Biotec) and IL‐15 (50 IU mL^−1^, Miltenyi Biotec).

For transduction assays, pre‐activated T cells were seeded at a density of 8 × 10^4^ cells per well in flat‐bottom 96‐well plates. Following LV inoculation, cells were centrifuged at 850×g, 32 °C, for 90 min, in the presence or absence of 30 µM rapamycin in 0.5% DMSO. Medium was completely refreshed post‐spinoculation removing residual rapamycin and cells were further cultured for 3–4 days before flow cytometry assessment. Co‐culture assays with tumor cells were performed in plain medium (TCM or 4Cell Nutri‐T medium) without cytokine supplementation. Alternatively to rapamycin, vectofusin‐1 (Miltenyi Biotec) was used as a transduction enhancer as described before.^[^ ^10^ ^]^

### Lentiviral Vector Generation and Titration

Receptor‐targeted lentiviral vectors were produced as described previously.^[^ ^38^ ^]^ In brief, HEK‐293T cells were transiently transfected with a plasmid cassette, comprising packaging plasmid (pCMV∆R8.9) and a transfer plasmid encoding either CD19‐CAR (pSEW‐mycCD19‐CAR‐28z) or green fluorescent protein (GFP) (pSEW‐GFP). For the generation of different LVs, we co‐transfected plasmids encoding the respective glycoproteins from vesicular stomatitis virus (pMD2.G) or Nipah targeting CD8α receptor (pCAGGS‐NiV‐Gd34‐CD8‐scFv, pCAGGS‐NiV‐Fd22) or measles targeting CD4 receptor (pCG‐Hmut‐CD4‐DARPin, pCG‐FcΔ30).^[^ ^9^, ^39^ ^]^ LVs were harvested and concentrated through centrifugation on a 20% sucrose cushion at 4500×g (4 °C), re‐suspended in Dulbecco's phosphate buffer saline (DPBS, Mg^2+^ and Ca^2+^ free), aliquoted and stored at −80 °C. The residual plasmid was removed from LV stocks with 1.5 U µL^−1^ DNase I (Thermo Fisher Scientific, Bremen, Germany) at 37 °C for 30 min. CD8‐LVs used for the in vivo studies were produced in HEK‐293T β2M knockout CD47^high^ (HEK‐293T β2M^−/−^, CD47^hi^) cells.^[^ ^40^ ^]^

Both VSV‐LV and CD8‐LV vectors were titrated on MOLT‐4.8 cells, while transduction units per mL (TU mL^−1^) for CD4‐LV were determined on A301 cells. LV particle number was measured by Nanosight NS300 (Malvern Panalytical) and used as the main value for normalization of different vector stocks wherever possible.^[^ ^41^ ^]^ See Table S2 (Supporting Information) for vector dosages applied in samples processed for scRNA‐seq.

### Next Generation Sequencing

Single cells were isolated in microwell cartridges and mRNA was extracted with the BD Rhapsody pipeline according to the manufacturer's instructions. In total, 5 cartridges were used. The 4 samples (Control, VSV‐LV, CD8‐LV, CD4‐LV) of donor 1 were separated into different cartridges, while the remaining 8 samples covering donors 2 and 3 were multiplexed in one cartridge (Table S3, Supporting Information). Whole transcriptome analysis was performed with NextSeq 550 (Illumina, San Diego, USA) or NextSeq 2000. Raw FASTQ sequencing files were processed and aligned to the human reference genome, supplemented with the sequence of the CD19‐CAR transgene, with the Seven Bridges Genomics software (Charlestown, MA, USA). Bioinformatic analysis of generated recursive substitution error correction (RSEC)‐adjusted molecule count matrices was performed in R Studio (R 4.2.1). Low‐quality cells of high mitochondrial and ribosomal gene content and other outliers as depicted in Figure S17A (Supporting Information) were filtered out with the quickPerCellQC function of the scuttle package and count matrices were normalized with the deconvolution method of the scran package and then log‐transformed.^[^ ^42^, ^43^ ^]^ Cell numbers per sample pre‐ and post‐filtering are shown in Table S2 (Supporting Information).

Further downstream analysis was conducted with the Seurat package.^[^ ^44^ ^]^ Transcriptional variation from the cell cycle was removed (Figure S17B, Supporting Information). Principal component (PC) analysis was performed, from which the first 22 PCs were chosen for the dimensionality reduction in Uniform Manifold Approximation and Projection (UMAP) plots (Figure S17C, Supporting Information). Batch effect correction was applied by integrating the donors, while technical effects due to different cartridge use were not observed (Figure S17D, Supporting Information). To isolate CD8, CD4, and CAR T cells, multimodal analysis was performed on CD8A and CAR gene expression as previously described with the multimode package (Figure S2, Supporting Information).^[^ ^11^ ^]^ Differential gene expression analysis was conducted with the FindMarkers algorithm using the Wilcoxon sum rank test. False discovery rate (FDR) by Benjamini and Hochberg ^[^ ^45^ ^]^ was calculated instead of Bonferroni p‐value adjustment and genes outside a range of |log_2_(fold‐change)| > 0.2 and FDR < 0.05 were considered as differentially expressed (DE). Volcano plots were constructed with the EnhancedVolcano package and gene set enrichment analysis was performed with the WebGestaltR or escape packages or Appyters online tool against gene sets from the Kyoto Encyclopedia of genes and genomes (KEGG) or hallmark collection.^[^ ^46^, ^47^, ^48^, ^49^, ^50^, ^51^ ^]^ Genes of interest are plotted in violin plots, on which FDR values from the respective differential gene expression analyses are shown.^[^ ^28^ ^]^ Numeric results of differential gene expression analyses are available upon request.

### Mouse Experiments

Animal experiments were formally approved by the institutional and national animal care committees (approval numbers: 107/1057 and 107/2007) and performed according to the German animal protection law and European Union guidelines. Female NSG mice (NOD.Cg.Prkdc^scid^IL2rg^tmWjl^/SzJ, Jackson Laboratory, CA, USA) were transplanted with 3‐day pre‐activated human PBMC (10^7^ cells per mouse in 100 µL DPBS) by intravenous injection. The following day, rapamycin (3 mg kg^−1^ or 8 mg kg^−1^), vehicle (2% DMSO, 30% PEG300, 5% Tween 80 in water), or PBS were intraperitoneally administered. One hour post‐administration, CD8‐LV was intravenously injected, accounting for 3.16 × 10^11^ particles per mouse in the case of GFP encoding LVs and 1.83 × 10^11^ particles per mouse for CD19‐CAR encoding LVs.

For the tumor model, mice were intravenously injected with 5 × 10^6^ Nalm‐6‐luc‐eBFP cells stably expressing firefly luciferase, in 100 µL DPBS, 6 days prior to LV injection. Health status (weight, posture, behavior) was monitored at least two times weekly. Tumor burden was weekly assessed 10 min after intraperitoneal injection of 150 mg kg^−1^ D‐luciferin (PerkinElmer, Rodgau, Germany) with the in vivo imaging system (IVIS, PerkinElmer) under anesthesia. Both in vivo experiments were conducted with PBMC isolated from the same donor and activated in 4Cell Nutri‐T medium as mentioned above. The chromosomal integration of the transgene in human T cells was quantified by vector copy number (VCN) assay via real‐time qPCR using genomic DNA, as described before.^[^ ^12^ ^]^

### Drug

For cell cultures, a final concentration of 30 µM rapamycin (Hölzel Diagnostika Handels GmbH, Cologne, Germany) was used during the transduction of PBMC. DMSO reached 0.5% in cell culture. For in vivo studies, concentrations of 0.75 and 2 mg mL^−1^ were formulated in 2% DMSO, 30% PEG300 (Sigma‐Aldrich), 5% Tween 80 (Sigma‐Aldrich), and water (Sigma‐Aldrich). Endotoxin assay was conducted for the vehicle and the two formulations with kinetic turbidimetric plate assay (Associates of Cape Cod Inc., MA, USA), detecting <0.1 EU mL^−1^.

### Western Blot

Pre‐activated PBMC treated for 1.5 h with increasing concentrations of rapamycin or 500 IU mL^‐1^ IFNα2β (BioLegend, CA, USA) were analyzed for IFITM1, IFITM2/3, and GAPDH protein expression. Cells were washed twice with PBS and lysed with RIPA buffer with protease inhibitors (cOmplete, Roche, Basel, Switzerland) for 10 min on ice at a density of 8  ×  10^3^ cells µL^‐1^, following 15 min centrifugation at 16000 × g. Supernatants were mixed with SDS loading dye (240 mM Tris‐HCl, 8% SDS, 40% glycerin, 0.2% bromophenol blue, 20% β‐mercaptoethanol, dH_2_O) and incubated at 95 °C for 10 min. Lysates from ≈2  ×  10^5^ cells were loaded into 12% SDS‐PAGE gels. Resolved proteins were transferred onto a PVDF membrane, which was blocked with 10% horse serum in TBS‐T buffer at room temperature for 1 h. Proteins of interest were stained with 1:1000 diluted anti‐IFITM1 (clone EPR22620‐12, Abcam, Cambridge, UK), anti‐IFITM2/3 (clone EPR5242, cross‐reactive with IFITM2 and IFITM3, Abcam) and anti‐GAPDH‐HRP (Abcam) in TBS‐T with 5% horse serum by incubating overnight at 4 °C. Anti‐rabbit‐HRP (Agilent Dako, Glostrup, Denmark) diluted 1:2000 was used accordingly. Chemiluminescence reaction was performed with Pierce ECL substrate (Thermo Fisher Scientific), visualization was carried out by MicroChemi device (DNR Bio‐Imaging systems, Neve Yamin, Israel), and protein quantification was performed with FiJi software, where percentages of areas were calculated. Fold changes were calculated by the formula: $Fold\;change\;=\;\frac{\%treated_{IFITM}\times \%ctrl_{GAPDH}}{\%treated_{GAPDH}\times \%ctrl_{IFITM}}$.

### Mass Spectrometry

Reversed‐phase liquid chromatography‐tandem mass spectrometry (LC‐MS/MS), using a semi‐automated version of the SP3 protocol,^[^ ^52^ ^]^ was performed with samples derived from 6 donors (1.5 × 10^7^ cells per condition per donor), which were treated either with 30 µM rapamycin or 0.5% DMSO (drug diluent) or left untreated in TCM medium supplemented with cytokines for 90 min at 37 °C. Extracted protein aliquots of 50 µg, adjusted to 95 µL, were digested as previously described.^[^ ^53^ ^]^ Each sample was analyzed in triplicates. LC‐MS/MS analysis was carried out using an Orbitrap Eclipse Tribid mass spectrometer (Thermo Fisher Scientific, Bremen, Germany) coupled to an UltiMate 3000 RSLCnano system equipped with an EASY‐Spray source (Thermo Fisher Scientific, Germering, Germany). Raw data were searched against the *Homo sapiens* Uniprot reference proteome database (downloaded 8th May 2021) and a contaminant sequence set provided by MaxQuant.^[^ ^54^ ^]^ Differential protein analysis was then carried out using Perseus v1.6.6. Data were log_2_‐transformed and missing values were imputed based on normal distribution. To reduce donor variation, data were normalized using a mean‐centering approach as described.^[^ ^55^ ^]^ Volcano plots were created in R with ggplot2. Extended protocol was described in the Supporting Information and in Table S4 (Supporting Information). The numeric results from differential protein expression analysis are available upon request.

### Ex Vivo Co‐Culture Assay

Splenocytes (2 × 10^5^) or bone marrow cells isolated from vector‐injected animals were cultured with 10^5^ irradiated (10 Gy) Nalm‐6 cells per well in NutriT medium with cytokines and antibiotics. The samples were cultured in technical duplicates in 96‐well plates and the expression of CAR was assessed by flow cytometry 7 days later. Technical replicates were averaged, and values were plotted and statistically analyzed.

### Sample Preparation and Flow Cytometry

For flow cytometry assays, blood, spleen, and bone marrow samples were collected from mice. In order to obtain a single‐cell suspension, spleens, and bone marrow extractions were gently pressed through a 70 µm cell strainer. Red blood cells were lysed with 1x BD Pharm Lyse buffer (BD Biosciences, NJ, USA) for 15 min at room temperature. Cells were stained for 30 min at 4 °C with the appropriate combination of the following antibodies: anti‐c‐myc[fluorescein] (SH1‐26E7.1.3, Miltenyi Biotec), anti‐CD3[BV605] (HIT3a, 564712, BD Biosciences) or anti‐CD3[PerCP] (BW264/56, Miltenyi Biotec), anti‐CD45[BV510] (2D1, BioLegend), anti‐CD8[BV786] (RPA‐T8, BD Biosciences) or anti‐CD8[VioBlue] (BW135/80 or REA734, Miltenyi Biotec), anti‐CD4[PE‐CF549] (RPA‐T4, BD Biosciences) or anti‐CD4[PE‐Vio770] (VIT4, Miltenyi Biotec), anti‐CD19[PE‐Vio770] (LT19, Miltenyi Biotec), anti‐TIM3[allophycocyanin] (REA635, Miltenyi Biotec), anti‐LAG3[phycoerythrin] (3DS223H, Thermo Fisher Scientific, MA, USA), anti‐PD‐1[PE‐Vio770] (PD1.3.1.3, Miltenyi Biotec), anti‐CD62L[PE‐Vio770] (145/15, Miltenyi Biotec), anti‐CD45RA[VioBlue] (T6D11, Miltenyi Biotec), anti‐CD45RO[allophycocyanin or PerCP] (UCHL1, Miltenyi Biotec), anti‐CCR7[APC] (REA546, Miltenyi Biotec) and with a fixable viability dye (eFluor780, Thermo Fisher Scientific). Mouse samples were blocked for 20 min at 4 °C with mouse and human Fc receptor blocking reagents (Miltenyi Biotec) prior to staining. Fluorescently labeled cells were measured at MACSQuant Analyzer 10 (Miltenyi Biotec) or LSRFortessa (BD Biosciences). Flow cytometry data were analyzed with either FCS Express v6 (De Novo Software, CA, USA) or with FlowJo v10 (BD Biosciences). Representative flow cytometry plots and gating strategies are shown in Figures S15A and S18 (Supporting Information), for the CD19‐CAR and GFP in vivo mouse studies, respectively.

### Statistical Analysis

Comprehensive information about pre‐processing and statistical analysis of the scRNA‐seq data is described above, under *Next generation sequencing*. Apart from scRNA‐seq data in none of the reported datasets outliers were excluded from data analysis. Exported numeric results were statistically analyzed and plotted with GraphPad Prism 9 (GraphPad Software Inc., CA, USA) or R environment. Mean values with standard deviation (SD) are shown in graphs or in text. Sample sizes (*n*) were selected such that statistically relevant results were expected and were provided in figure legends. Statistical differences were assessed with two‐tailed paired or unpaired t‐test, one‐way or two‐way ANOVA with Tukey's multiple comparisons test, and the significance was shown on graphs either with a number or with the following abbreviations: ns = non‐significant, ∗*p<* 0.05, ∗∗*p <* 0.01, ∗∗∗*p <* 0.001, ∗∗∗∗*p <* 0.0001. Mouse tumor luminescence data were log‐transformed prior to two‐way ANOVA analysis.

## Conflict of interest

C.J.B. is listed as a co‐inventor of the CD8‐targeted lentiviral vector patent. All other authors declare no conflicts of interest.

## Author Contributions

F.T.C. and C.J.B. conceptualized the project. Methodology involved F.T.C., N.H., E.A., A.H.B., C.T., L.S., and F.B.T. C.J.B., C.C., J.B., F.B.T., and L.C. handled validation. Formal analysis was done by F.T.C. and L.S. Investigation tasks were performed by F.T.C., N.H., A.H.B., E.A., and C.T. Resources were managed by C.J.B. and F.B.T. Data curation was F.T.C.'s responsibility. Writing – original draft was done by F.T.C. and C.T. C.J.B. and N.H. handled writing review and editing. Visualization was done by F.T.C. C.J.B. supervised the project and managed project administration. Funding acquisition involved C.J.B., J.B., and C.C.

## Supporting information

Supporting Information

## Data Availability

The data described in this publication have been deposited in NCBI's Gene Expression Omnibus (GEO) and are accessible through GEO Series accession number GSE228824. (https://www.ncbi.nlm.nih.gov/geo/query/acc.cgi?acc=GSE228824).^[^ ^56^ ^]^ Raw sequencing files are deposited in Sequence Read Archive (SRA) under the project number PRJNA950024. The R markdown code used for the current analysis is available in the GitHub public repository (https://github.com/FilipCha/scRNA‐seq‐CAR‐T‐cell‐analysis.git).
