# Supplementary material for: CAR Gene Delivery by T‐cell Targeted Lentiviral Vectors is Enhanced by Rapamycin Induced Reduction of Antiviral Mechanisms
Source: Adv Sci (Weinh). 2023 Oct 30;10(35):2302992. doi: 10.1002/advs.202302992 (PMC10724389; doi:10.1002/advs.202302992)
Supplement: Supplementary file 1 — Supporting Information [file ADVS-10-2302992-s001.pdf]

ADVANCED SCIENCE
Open Access

Supporting Information

for *Adv. Sci.*, DOI 10.1002/advs.202302992

CAR Gene Delivery by T-cell Targeted Lentiviral Vectors is Enhanced by Rapamycin Induced Reduction of Antiviral Mechanisms

*Filippos T Charitidis, Elham Adabi, Naphang Ho, Angela H Braun, Ciara Tierney, Lisa Strasser, Frederic B Thalheimer, Liam Childs, Jonathan Bones, Colin Clarke and Christian J Buchholz**

Supplementary Material

CAR gene delivery by T-cell targeted lentiviral vectors is enhanced by rapamycin induced reduction of antiviral mechanisms

Filippos T Charitidis[1], *Elham Adabi*[1], *Naphang Ho*[1], *Angela H Braun*[1,2], *Ciara Tierney*[3], *Lisa Strasser*[3], *Frederic B Thalheimer*[1,4], *Liam Childs*[5], *Jonathan Bones*[3,6], *Colin Clarke*[3,7], *Christian J Buchholz*[1,2,4]

[1] Molecular Biotechnology and Gene Therapy, Paul-Ehrlich-Institut, 63225 Langen, Germany.

[2] Deutsches Krebsforschungszentrum and German Cancer Consortium (DKTK), 69120 Heidelberg, Germany.

[3] Characterisation and Comparability Laboratory, National Institute for Bioprocessing Research and Training, Foster Avenue, Mount Merrion, Blackrock, A94 X099 Co. Dublin, Ireland.

[4] Frankfurt Cancer Institute (FCI), Goethe University, 60590 Frankfurt am Main, Germany.

[5] Host-Pathogen Interactions, Paul-Ehrlich-Institut, 63225 Langen, Germany.

[6] School of Chemical and Bioprocess Engineering, University College Dublin, Belfield, D04 V1W8 Dublin 4, Ireland.

[7] National Institute for Bioprocessing Research and Training, Fosters Avenue, Mount Merrion, Blackrock, A94 X099 Co. Dublin, Ireland.

Correspondence: Christian J. Buchholz, Paul-Ehrlich-Strasse 51-59, 63225 Langen (Hessen), Germany. Christian.Buchholz@pei.de

Mass spectrometry – Extended methodology

Samples were washed twice with cold PBS, re-suspended in 200 µL RIPA buffer with protease inhibitors (cOmplete™, Roche), sonicated for 5 min in an ultrasonic bath (Bandelin, Berlin, Germany) at 4°C, centrifuged at 16,000×g and supernatants were collected. Following cell lysis, protein concentration was determined using Pierce™ 660nm Protein Assay Kit (Thermo Fisher Scientific, Dublin, Ireland), as per the manufacturer's guidelines. Proteins in 50 µg aliquots were reduced and alkylated followed by a clean-up using magnetic beads and tryptic digestion over 4 hours. Following digestion, magnetic beads were removed, and samples were ready for LC-MS/MS. Each sample was analyzed in triplicates using an Orbitrap Eclipse™ Tribid™ mass spectrometer (Thermo Fisher Scientific, Bremen, Germany) coupled to an UltiMate™ 3000 RSLCnano system equipped with an EASY-Spray™ source (Thermo Fisher Scientific, Germering, Germany). Using a flow rate of 20 µL min^{-1}, 1 µg of sample was loaded onto a PepMap C18, 5 µm, 5 mm × 30 µm trap column (Thermo Fisher, Sunnyvale, CA, USA). This was followed by separation utilizing an Easy-Spray PepMap RSLC C18 column, 2 µm, 75 µm × 50 cm (Thermo Fisher). Separation of tryptic peptides was performed at a flow rate of 250 nL min^{-1} with a column temperature of 45°C using a gradient of (A) 0.10% (v/v) formic acid in water and (B) 0.10% (v/v) formic acid in acetonitrile (Optima™ LC/MS grade, Fisher Scientific, Dublin, Ireland). A 2.5-hour long gradient was applied with conditions of 5% B for 5 min, followed by a linear gradient from 5 to 25% over 95 min. An increase to 35% B over 20 min was followed by 2 washes at 90% B for 5 min. Column re-equilibration was carried out at 5% B for 15 min. The Orbitrap Eclipse™ Tribid™ mass spectrometer was operated in data dependent, positive ion mode with an Orbitrap resolution setting of 120,000 (at m/z 200). RF lens was set to 50%, spray voltage was set to 1800 V and source temperature was maintained at 300°C. MS1 full scans were conducted at a scan range of m/z 200-2000. The automatic gain control (AGC) target was set to standard with automatic control of fill time. The 20 most abundant precursor ions were selected for fragmentation. HCD fragmentation was set to a collision energy of 28%, with an Orbitrap resolution setting of 30,000 (at m/z 200) for fragment scans. For MS2 scans, the AGC target was 200% and fill time control was set to automatic using an isolation window of 1.2 m/z and only charge states of +2 to +6 were included. An intensity threshold of 5×10^4 was applied and the dynamic exclusion was set at 60 sec, with ±5 ppm tolerance. Protein identification and label-free quantification (LFQ) was carried out using Proteome Discoverer 2.5 (Thermo Fischer Scientific). Raw data were searched against the *Homo sapiens* Uniprot reference proteome database (downloaded 8th May 2021) and a contaminant sequence set provided by MaxQuant.[33] A Sequest HT search was carried out using Percolator, with a minimum and maximum peptide length set to 6 and 144, respectively, a strict target FDR of 0.01 and a relaxed target FDR of 0.05. Trypsin was set as enzyme with a maximum of 2 missed cleavages. Precursor mass tolerance was set to 10 ppm and a fragment mass tolerance of 0.02 Da. Carbamidomethylation was set as static modification and dynamic modifications oxidation and N-terminal acetylation were used within the search criteria. Prior to further statistical analysis, data were filtered for contaminants and only protein identifications with a Sequest score > 0 were allowed. Additionally, data were filtered to contain at least 70% valid values per treatment group.

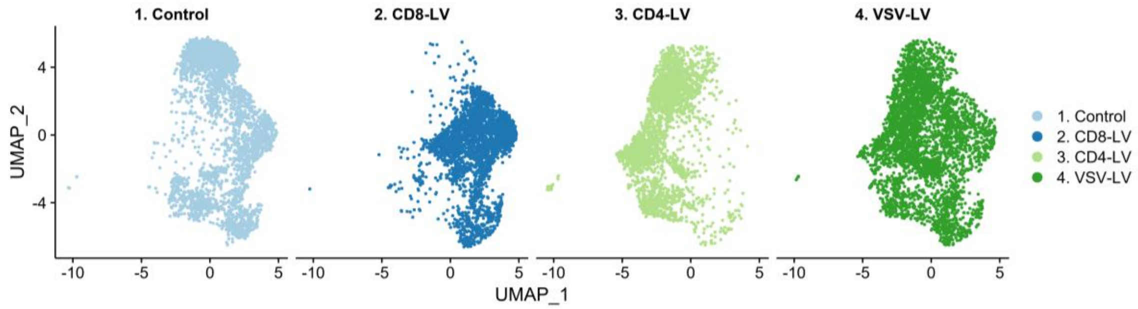

Figure S1: UMAP plot split by samples.
Control, CD8-LV, CD4-LV or VSV-LV inoculated PBMC samples (donors=3) plotted separately with the same UMAP coordinates.

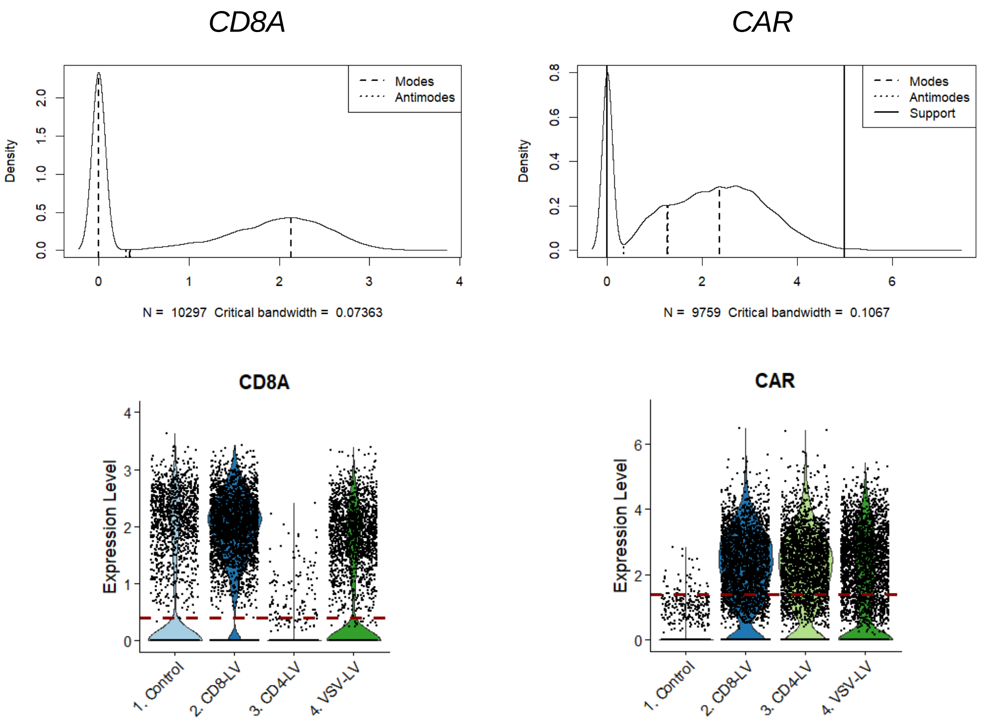

Figure S2: Multimodal analyses of CD8A and CAR gene expression.

Multimodal analyses (top) and violin plots (bottom) for setting the expression thresholds to subset CD8+ and CD4+ cells based on *CD8A* expression (left) and *CAR*- and *CAR*+ cells based on *CAR* expression (right). Dashed lines in violin plots indicate the thresholds.

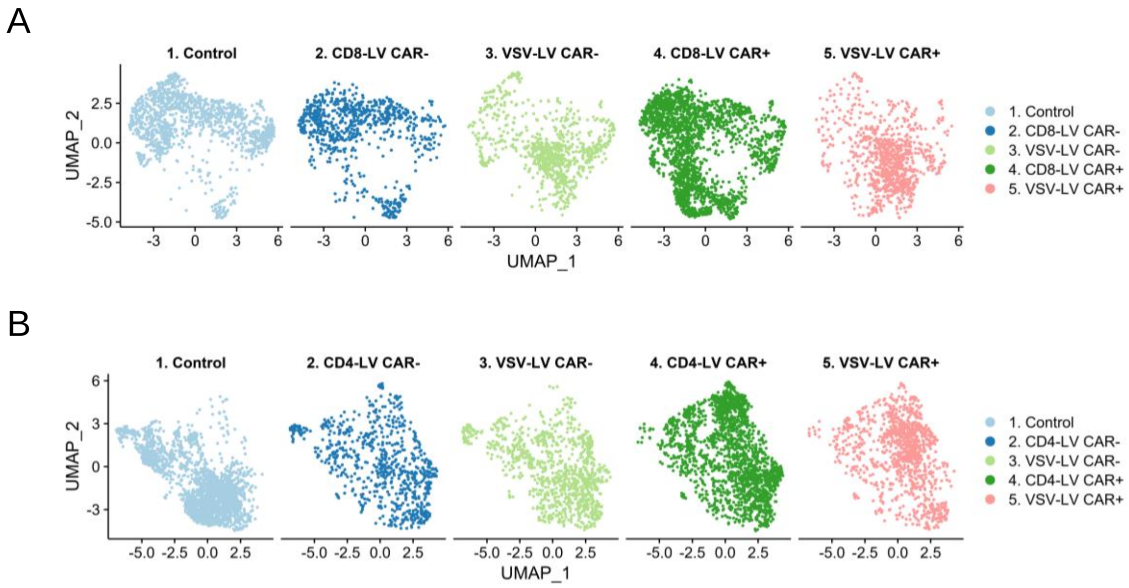

Figure S3: UMAP plots split by subsets.

(**A**) UMAP plot of CD8 cells split by selected subsets. (**B**) UMAP plot of CD4 cells split by subsets.

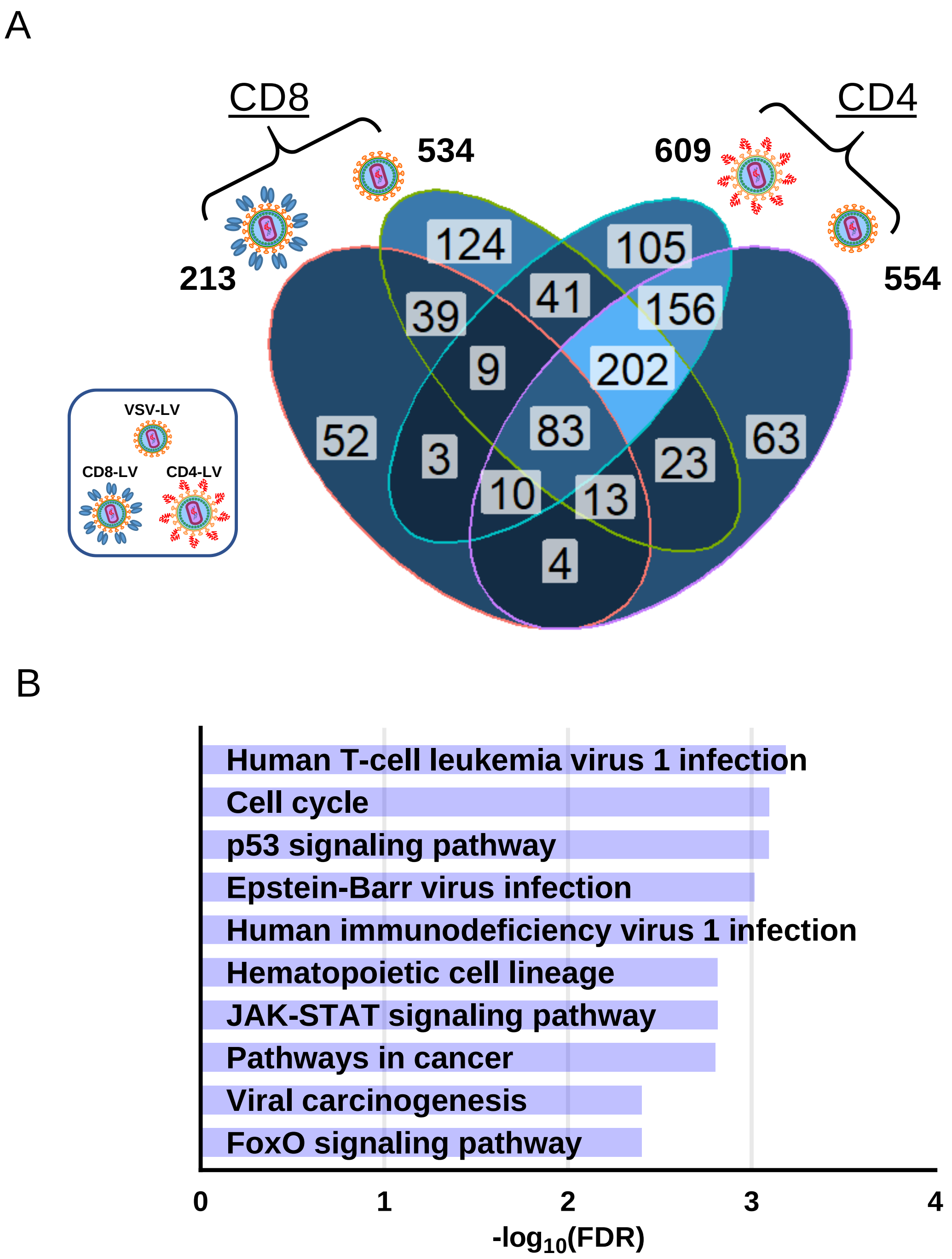

Figure S4: Diffential gene expression analysis of CAR+ and CAR- cells.
(**A**) Venn plot of shared DEGs ($|\log_2 FC| > 0.2$, $FDR < 0.05$) comparing LV-inoculated T cell types with the respective control cells. (**B**) Over-representation analysis of the 82 common genes from the diagram above on KEGG pathway gene sets.

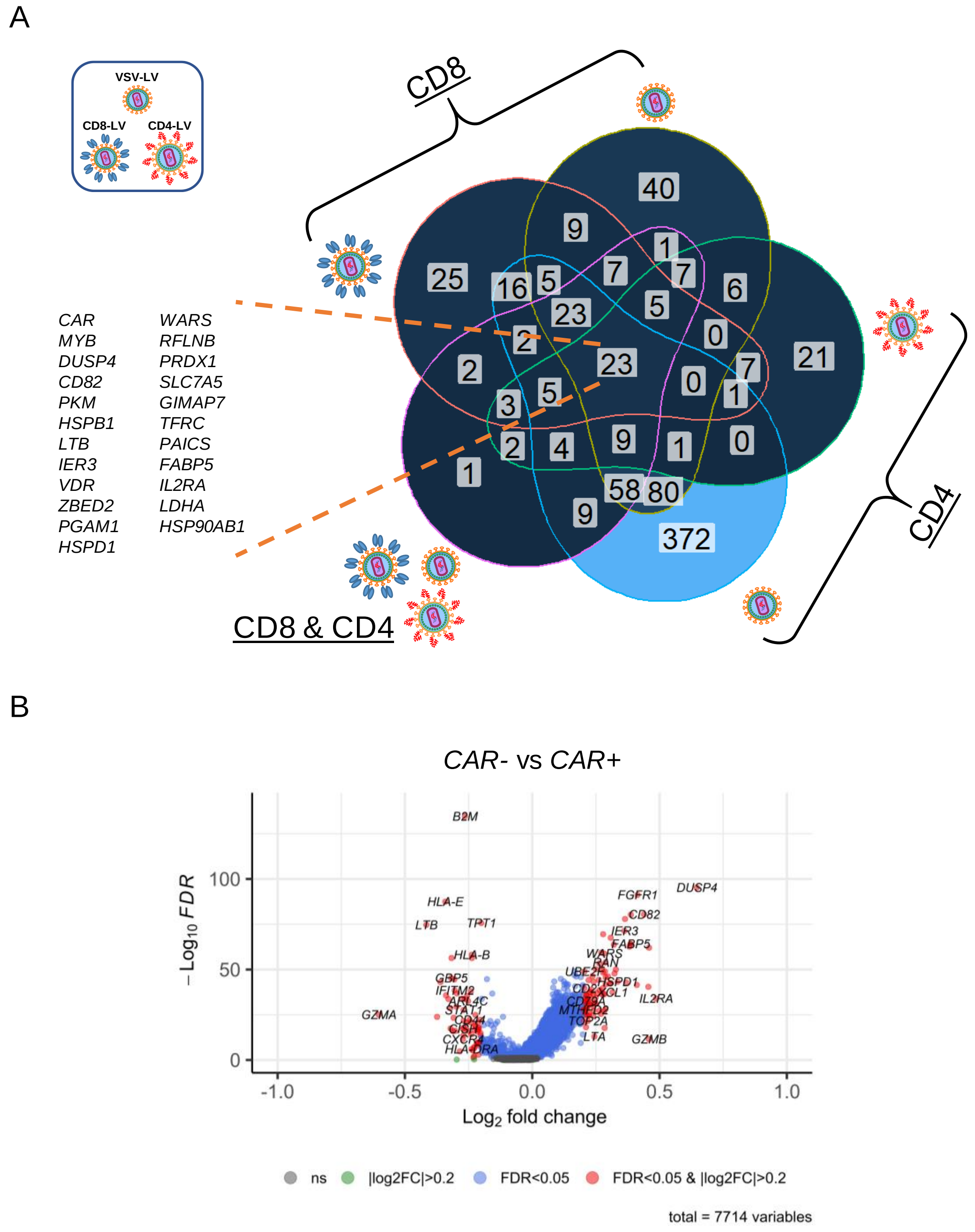

Figure S5: Diffential gene expression analysis of CAR+ and CAR- cells

(**A**) Venn plot of shared DEGs (|$\log_2$FC| > 0.2, FDR < 0.05) among CD8 and CD4 T cells exposed to the three different vector types as well as concatenated cell types and viral vectors, between *CAR*+ and *CAR*- cells. The 24 common genes between all groups are listed. (**B**) Volcano plot of DEGs comparing *CAR*+ and *CAR*- cells of all samples concatenated together (CD8-LV, CD4-LV, VSV-LV, both CD4 and CD8 cells).

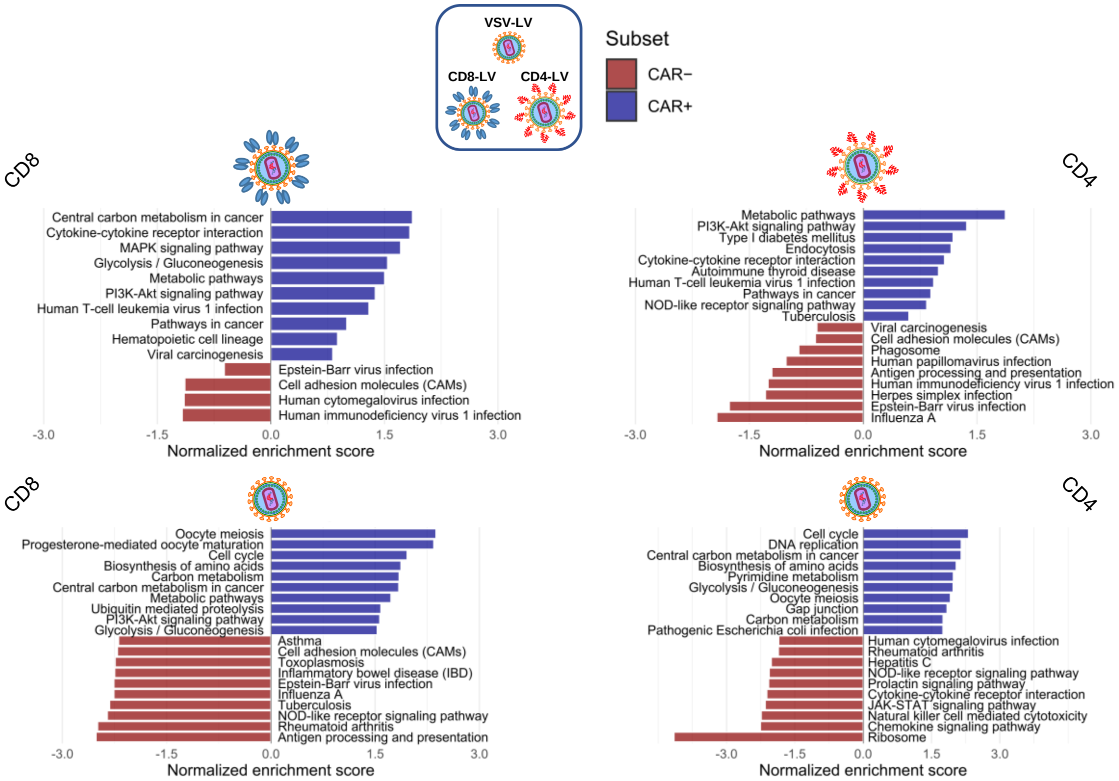

Figure S6: Gene set enrichment analyses of individual comparisons

KEGG pathway analysis of DEGs between *CAR*+ and *CAR*- cells of each individual comparison in CD8-LV, CD4-LV and VSV-LV samples, separating CD8 and CD4 cells ($|\log_2 FC|>0.2$, FDR < 0.05).

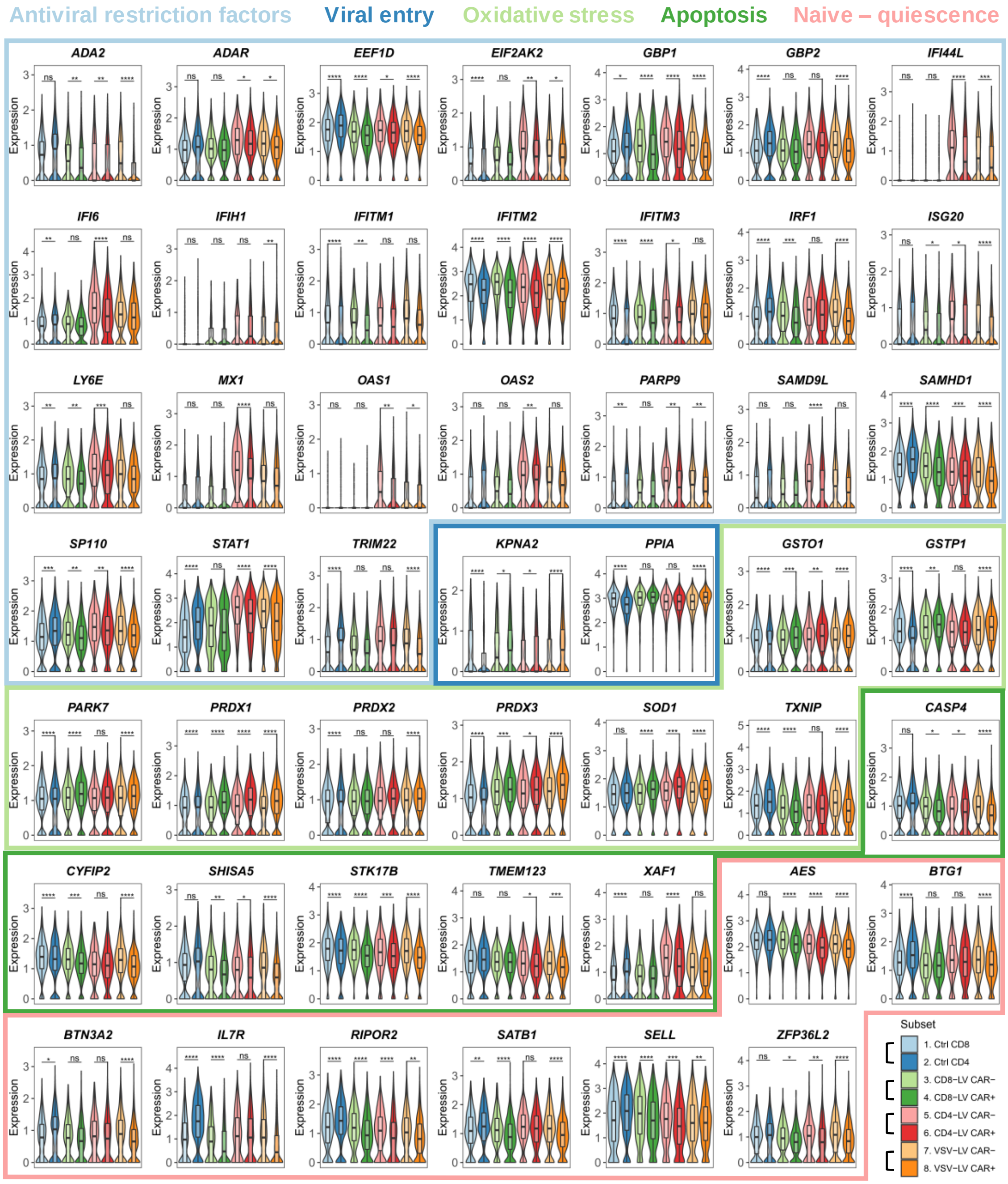

Figure S7: Violin plots of biologically relevant differentially expressed genes across subsets. Identified genes by differential gene expression analysis plotted for all analyzed subset cells and control cells (Wilcoxon rank sum test, $|\log_2 FC| > 0.2$, FDR < 0.05). Genes are alphabetically sorted and grouped into clusters associated with a biological function or state. ns: non-significant, *p<0.05, **p<0.01, ***p<0.001, ****p<0.0001.

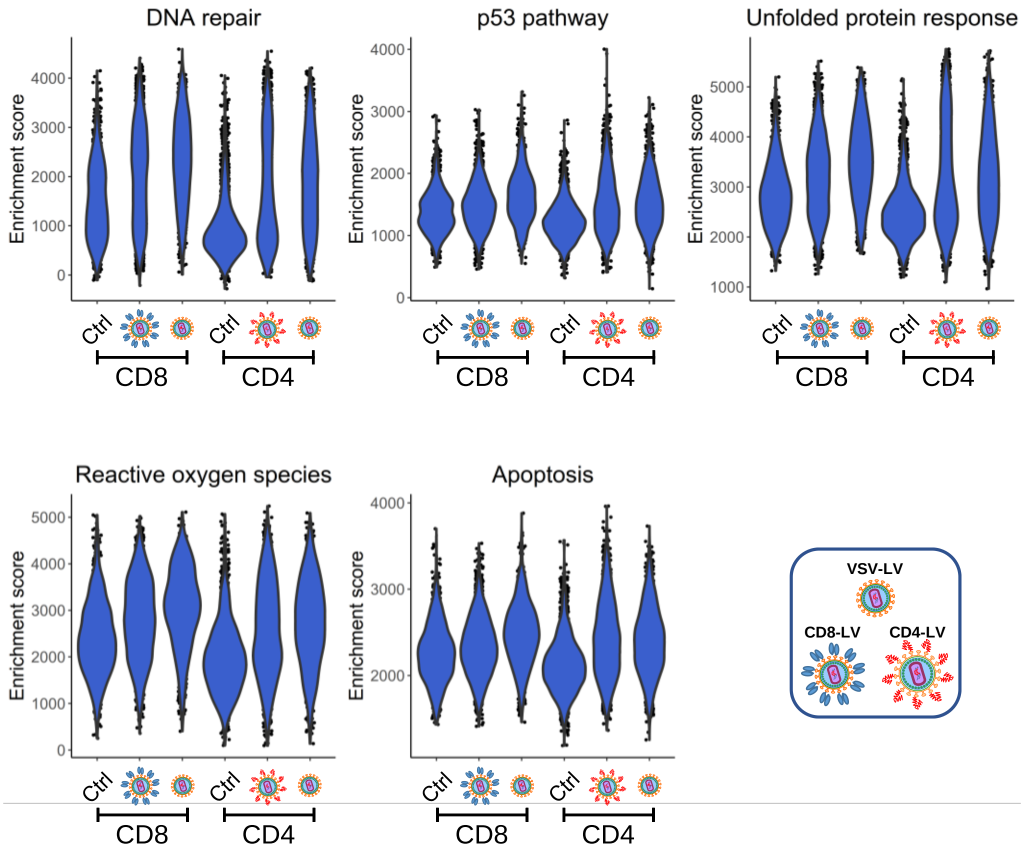

Figure S8: Gene set enrichment analysis of CAR T cells for stress-related pathways.
Violin plots of enrichment analysis for stress-related hallmark gene set collections in control and CAR T cells generated by VSV-LV, CD8-LV or CD4-LV. Except the control, only CAR-positive T cells were included in the analysis.

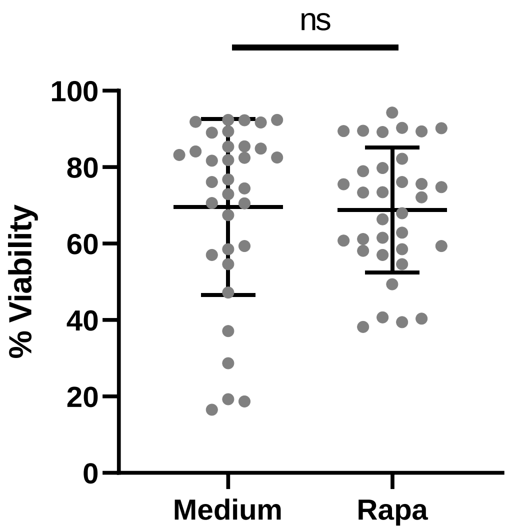

Figure S9: Viability of T cells incubated with or without rapamycin.
Data refer to Figure 3B and 3C. T cell viability determined by flow cytometry. Pre-activated T cells from different donors were incubated with plain medium or medium containing 30 μM rapamycin for 1.5 hour during spinfection with VSV-LV, CD8-LV or CD4-LV and processed 3 days later (n=33). Statistical analysis was conducted with unpaired t-test. Mean values with standard deviation are shown. ns: non-significant.

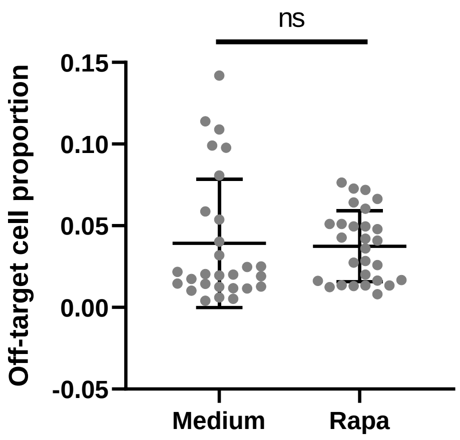

Figure S10: *In vitro* off-target gene delivery.
Proportion of *in vitro* off-target transduced cells out of total transduced human T cells for both, CD8-LV and CD4-LV, with or without 30 μM rapamycin (n=28). Off-target cells were gated as CD8-/CD3+/CAR+ for CD8-LV and CD8+/CD3+/CAR+ for CD4-LV. Transduction efficiency data are shown in Figure 3B. For statistical analysis unpaired t-test was performed and mean with standard deviation are shown. ns: non-significant.

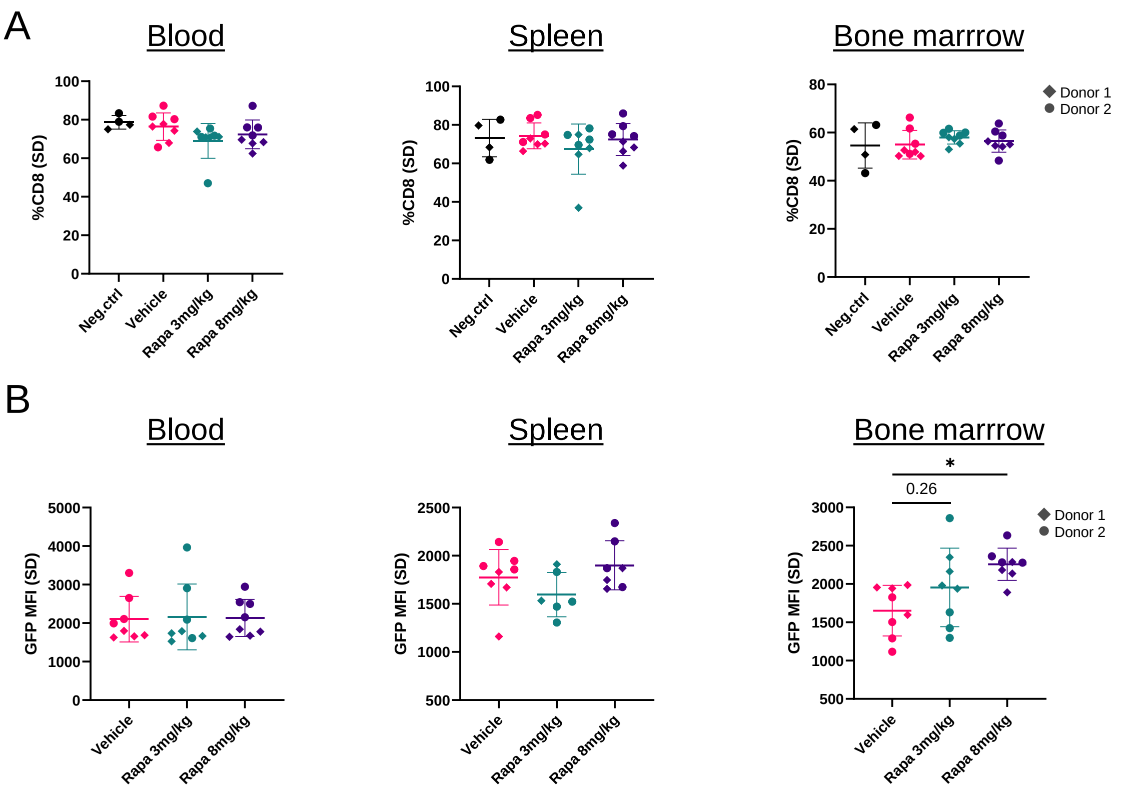

Figure S11: Supplemental data for the mouse study in Figure 5.
(**A**) Frequencies of human CD8+ T cells determined as CD4- out of hCD3+/hCD45+ cells by flow cytometry in blood, spleen and bone marrow (n=4-8). (**B**) Mean fluorescence intensities (MFI) of GFP expression of *in vivo* transduced human CD8+ cells.

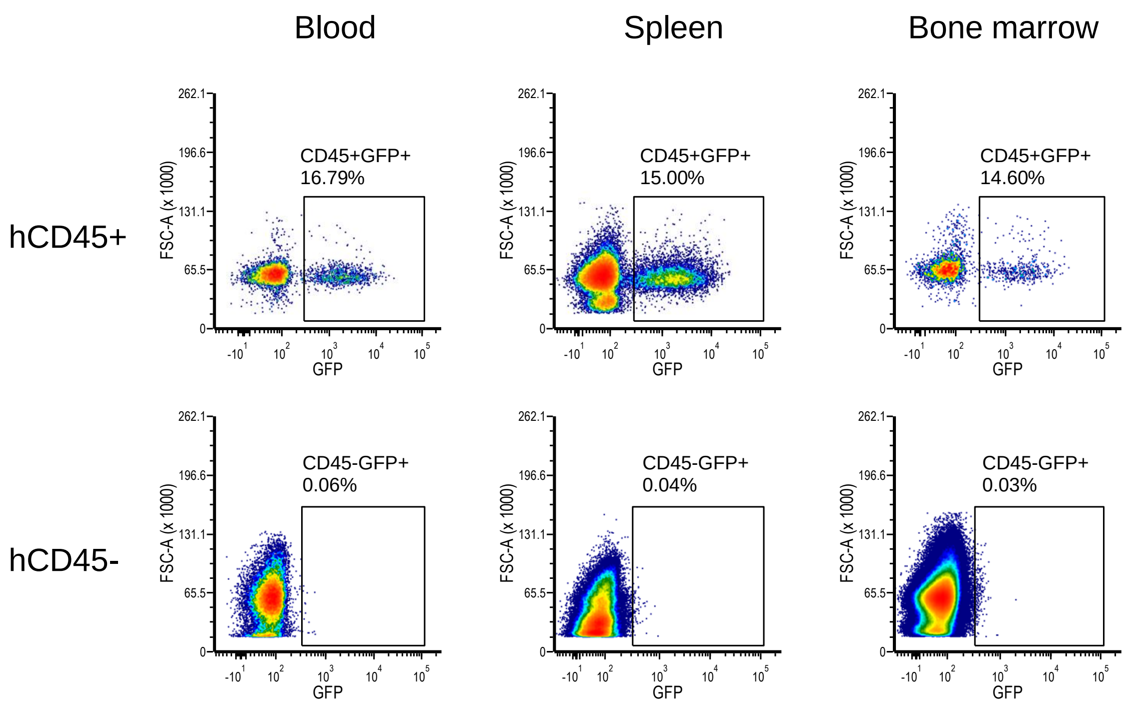

Figure S12: *In vivo* GFP transfer into human CD45 positive and negative (murine) cells. Supplemental data to Figure 5. Representative flow cytometry plots of human CD45+ (top panel) and total CD45- cells (bottom panel) harvested from blood, spleen and bone marrow of a rapamycin-treated mouse injected with GFP-transferring CD8-LV.

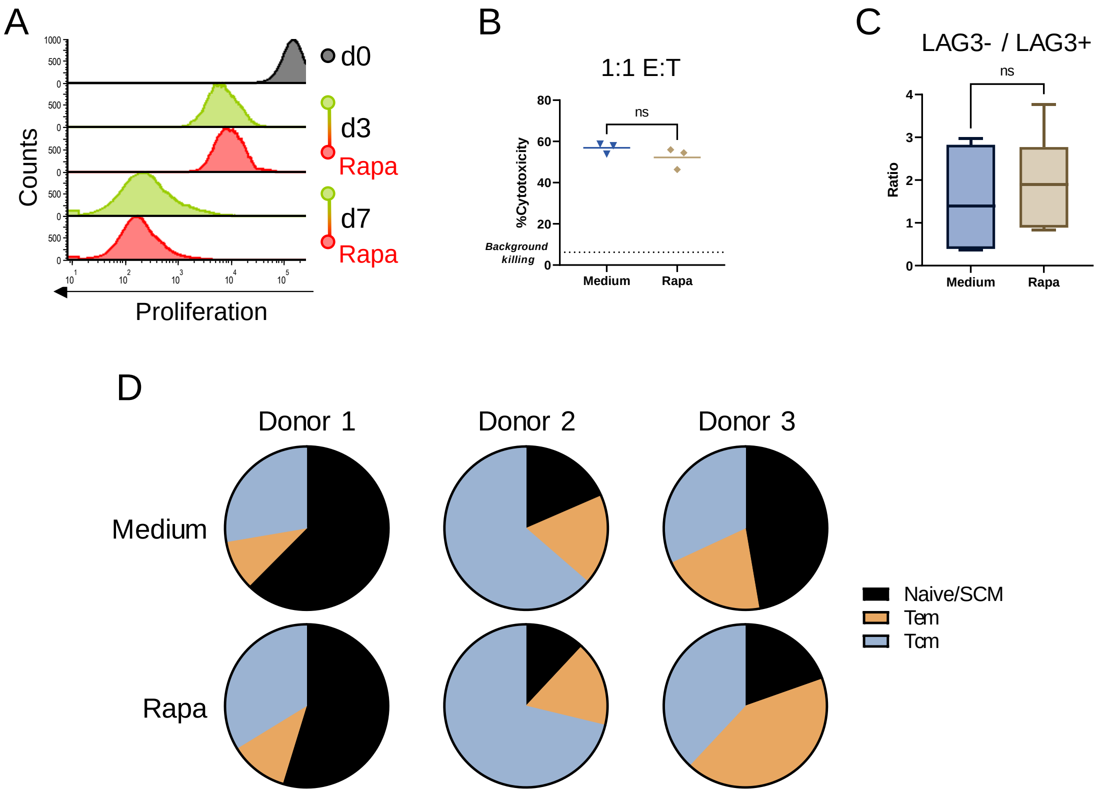

Figure S13: Effect of rapamycin on CAR expression, proliferation and CAR T cell phenotypes

(**A**) Proliferation of CD8+/CAR+ T cells generated by CD8-LV in presence or absence of rapamycin (donor=1). Pre-activated T cells were inoculated with CD8-LV on day 0 and CD8+/CAR+ T cells were assessed on days 3 and 7. (**B**) Cytotoxicity of 3-day expanded CD8 CAR T cells generated with or without rapamycin co-cultured with Nalm-6 tumor cells in 1:1 effector to target ratio and assessed 4 hours later (n=3). Total T cell number was normalized with control T cells cultured with or without rapamycin. Dotted line indicates the background killing mediated by control cells co-cultured with tumor cells. (**C**) Ratio of CD8+/CAR+/LAG3- to CD8+/CAR+/LAG3+ cells generated in presence or absence or rapamycin, on day 3 (n=11, donors=3). Whiskers indicate minimum and maximum value, box marks the 25th and 75th percentile and vertical line shows the median value. (**D**) Pie charts representing frequencies of memory phenotypes of CD8 CAR T cells of 3 donors generated in presence or absence of rapamycin, on day 3 (n=11, donors=3, SCM: stem cell memory). Statistical analysis was performed with unpaired t-test. ns: non-significant.

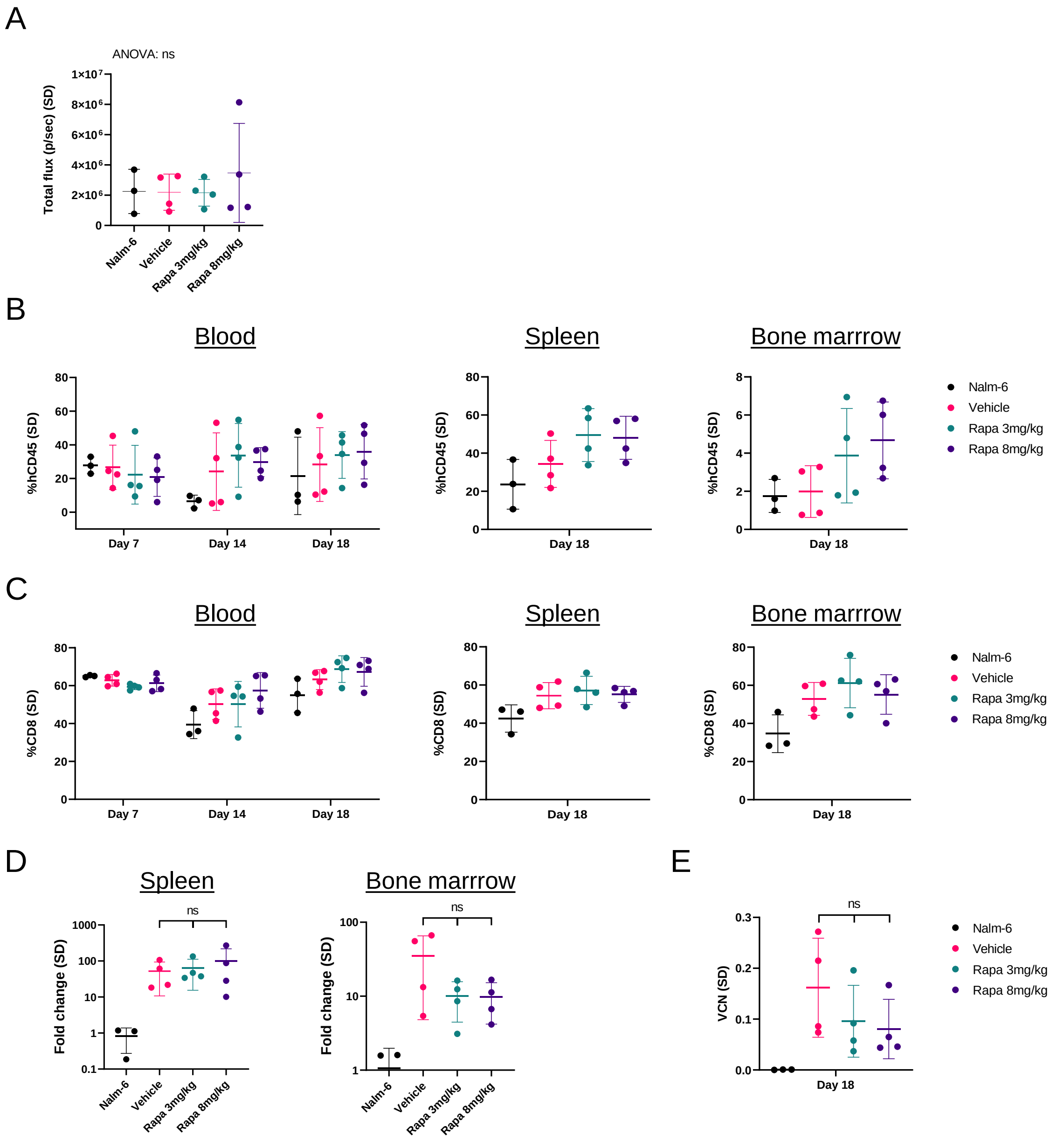

Figure S14: Supplemental data for the *in vivo* CAR tumor mouse study of Figure 6.
(**A**) Randomization of mice into groups based on the tumor burden determined by luminescence imaging of luciferase activity of engrafted Nalm-6 cells on day -2 prior CD8-LV injection. One-way ANOVA test was performed in log-transformed data (n=3-4). Result is indicated in the plot. (**B**) Humanization of mice defined as frequency of human CD45+ cells detected by flow cytometry among experimental groups on day 7, 14 and 18 in blood and on day 18 in spleen and bone marrow after CD8-LV injection. (**C**) Frequency of human CD8+ T cells out of hCD3+/hCD45+ cells in blood, spleen and bone marrow. (**D**) *Ex vivo* CAR T cell expansion from spleen and bone marrow samples (day 18) by co-culturing with irradiated Nalm-6 cells. Flow cytometry analysis was performed after 7 days of co-culture, cells from each mouse were seeded in technical duplicates, which were averaged before calculations. Fold changes were calculated for each individual mouse as ratio of the CD8+/CAR+ frequencies after co-culture and at the termination of the *in vivo* experiment. (**E**) Vector copy number (VCN) of CAR transfer gene integration in genomic DNA of human CD3+ cells sorted from mouse spleens. One-way ANOVA with Tukey's multiple comparisons was conducted. ns: non-significant.

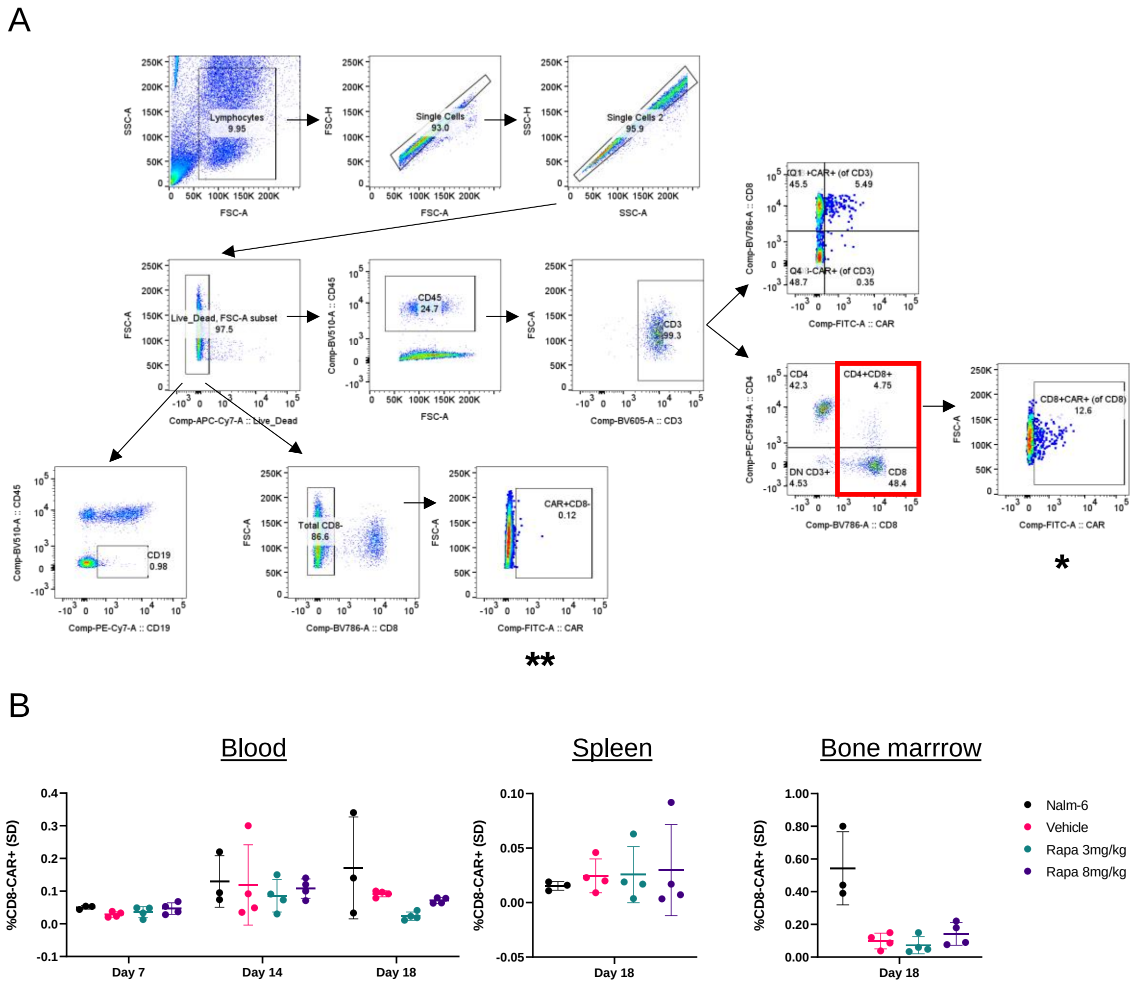

Figure S15: Off-target gene transfer after *in vivo* CD19-CAR delivery.
Data refer to Figure 6. (**A**) Gating strategy and representative plots of a blood sample from a mouse of the 8 mg kg^{-1} rapamycin group. Flow of gating steps are labeled by arrows; black frames indicate the subpopulation taken forward. The plot showing CAR+ cells of CD8+ and CD8+/CD4+ double positive human cells (red box) is labeled by a single asterisk, the plot showing the frequency of off-target CAR+ cells out of total CD8- human and murine cells is labeled by two asterisks. (**B**) Frequencies of *in vivo* off-target CAR+ cells out of total human and murine cells harvested from blood, spleen and bone marrow tissues. Mice having received only tumor cells but not vector particles (Nalm-6) provide the detection limit.

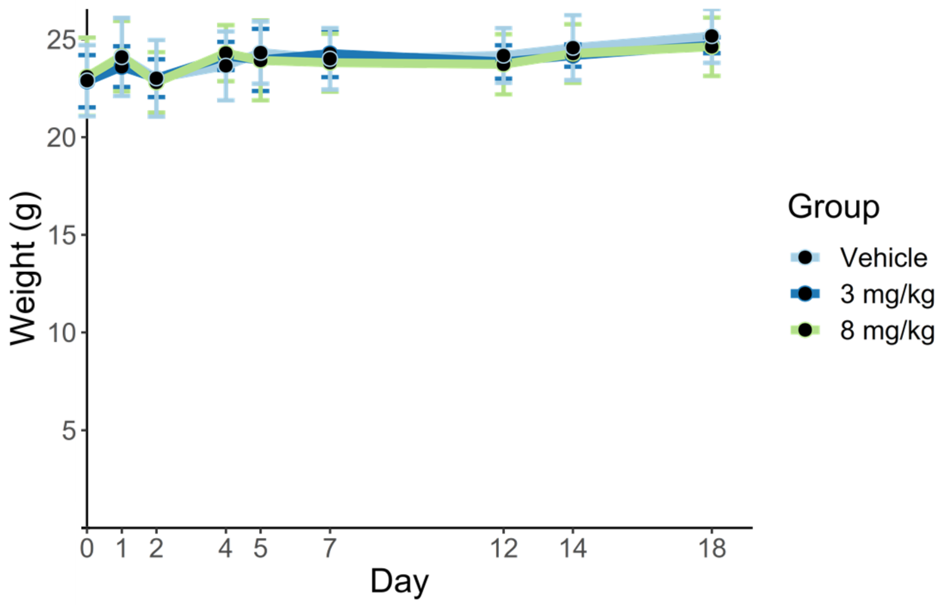

Figure S16: Animal weight monitoring.
Average weight with standard variation of all mice injected i.p. with vehicle, 3 mg kg^{-1} or 8 mg kg^{-1} rapamycin on day 0, from both, GFP (Figure 5) and CD19-CAR (Figure 6), *in vivo* studies.

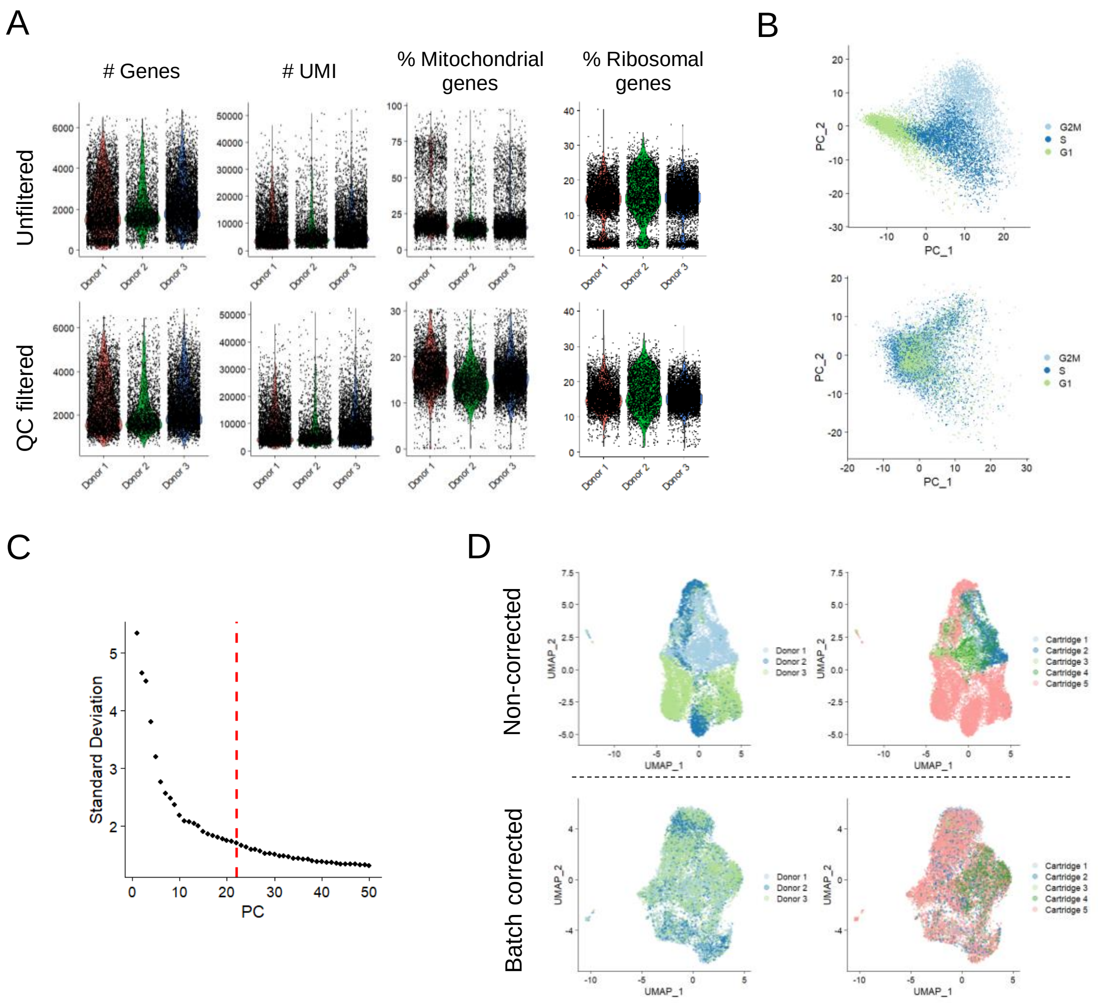

Figure S17: Pre-processing and batch effect correction of scRNA-seq data.
(**A**) Filtering out low quality cells based on the distribution of genes per cell, unique molecular indexes (UMIs) and frequencies of mitochondrial and ribosomal genes. (**B**) Principal components before (above) and after (below) cell cycle correction. (**C**) Elbow plot for selecting first 22 principal components for constructing the UMAP plots. (**D**) Donor effect (left) and technical effect (right), before (top) and after integrating the donors with Seurat (bottom).

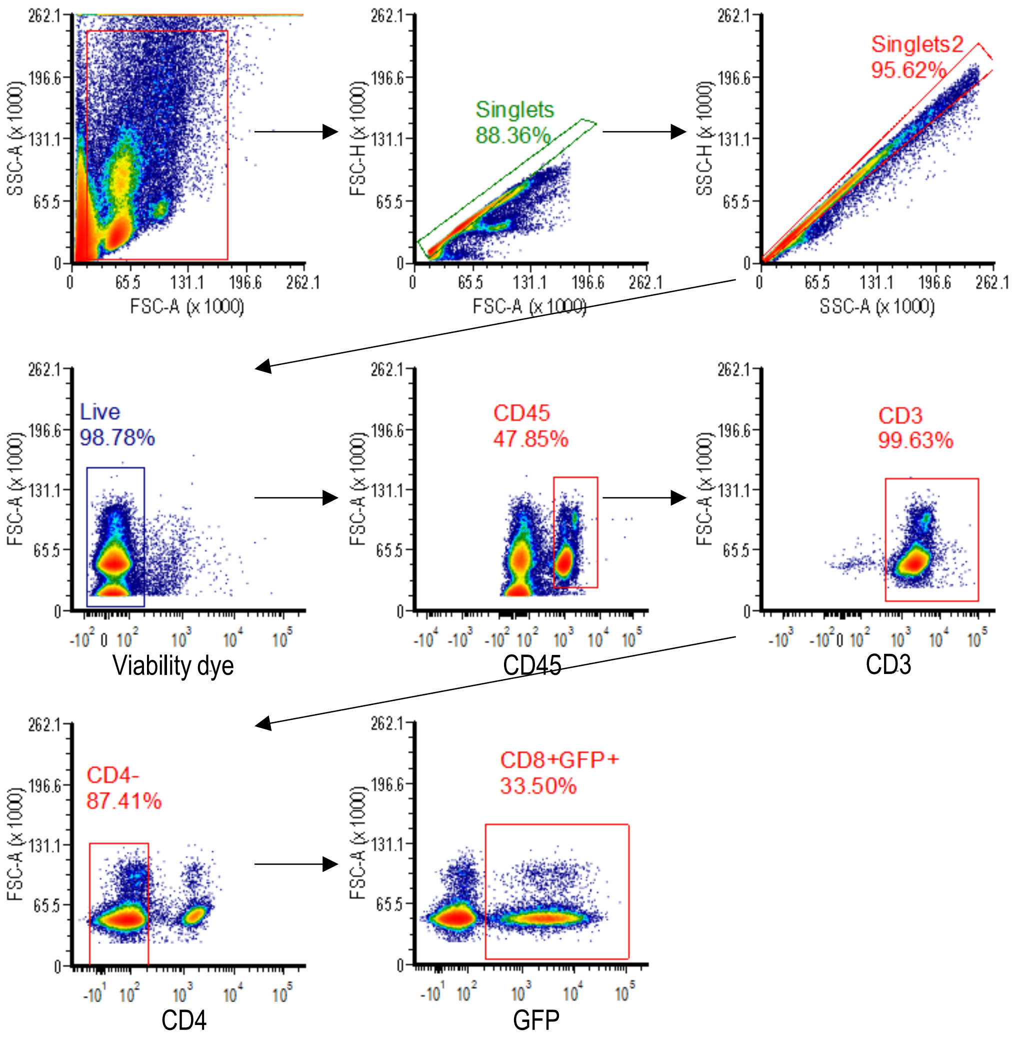

Figure S18: Gating strategy for flow cytometry analysis of *in vivo* GFP study.
Gating strategy and representative plots from a blood sample of a mouse having received CD8-LV and vehicle (Figure 5). Flow of gating steps are labeled by arrows; frames indicate the subpopulation taken forward. CD8+ target cells were gated as human CD4-/CD45+/CD3+ cells.

Table S1: On-target specificity of *in vivo* GFP delivery with CD8-LV‡.

		Blood %	Spleen %	Bone marrow %
Vehicle	Mean*	99.47	98.81	99.29
	SD	0.40	0.81	0.80
Rapamycin groups†	Mean#	99.01	99.09	98.88
	SD	1.59	0.43	0.76

‡On-target specificity determined as human CD45+/CD3+/CD4-/GFP+ cells †Refers to both 3 mg kg^{-1} and 8 mg kg^{-1} rapamycin groups.
*n=8 animals for each tissue.
#n=16 blood; n=13 spleen; n=16 bone marrow.

Table S2: GFP-positive murine cells from *in vivo* GFP delivery study with CD8-LV‡.

		Blood %	Spleen %	Bone marrow %
Neg.ctrl	Mean°	0.068	0.073	0.005
	SD	0.079	0.078	0.006
Vehicle	Mean*	0.041	0.081	0.005
	SD	0.040	0.103	0.005
Rapamycin groups†	Mean#	0.040	0.053	0.006
	SD	0.056	0.062	0.006

‡ Frequency of GFP+ cells calculated on hCD45-/hCD3- live cells.
†Refers to both 3 mg kg^{-1} and 8 mg kg^{-1} rapamycin groups.
°n=4 animals for each tissue.
*n=8 animals for each tissue.
#n=16 blood; n=13 spleen; n=16 bone marrow.

Table S3: Characteristics of the applied vectors, generated CAR T cells and processed cells for scRNA-seq analysis.

Sample	Particles / cell	VCN*	CAR %◊	scRNA-seq	
				**Pre-processed cells**	**Post-processed cells**
Control	-	n.d.⌖	-	4022	3419
CD8-LV	3163	1.2 ± 0.8	D1: 62.8%<br>D2: 13.1%<br>D3: 24.8%	3422	2978
CD4-LV	4419	1.8 ± 1.3	D1: 50.4%<br>D2: 6.6%<br>D3: 14.2%	3243	2881
VSV-LV	272	2.0 ± 0.2	D1: 64.4%<br>D2: 22.6%<br>D3: 54.4%	4647	3900

*VCN was measured in at least 2 technical replicates from the original post-sorted samples of each donor. Due to low sample size of one donor after sorting, DNA from the CD8-V generated sample was not recovered (control n=6, CD8-LV n=4, CD4-LV n=6, VSV-LV n=6) (mean ± standard deviation).

⌖Non-detectable.

◊CAR T cell frequency out of target cells (D1=donor 1, D2=donor 2, D3=donor 3).

Table S4: Samples and donors distribution to cartridges

Cartridge	Donor	Sample
1	1	Control
2	1	VSV-LV
3	1	CD8-LV
4	1	CD4-LV
5	2 & 3	All samples multiplexed

Table S5: Detailed description of (A) liquid chromatography gradient and (B) parameter values used for mass spectrometry and (C) Proteome Discoverer searches.

A)

Time (min)	Flow (µL min^{-1})	%B	Curve
0.000	0.250	5.0	5
5.000	0.250	5.0	5
100.000	0.250	25.0	5
120.000	0.250	35.0	5
121.000	0.250	90.0	5
126.000	0.250	90.0	5
126.100	0.250	5.0	5
130.000	0.250	90.0	5
135.000	0.250	90.0	5
135.100	0.250	5.0	5
150.000	**Stop Run**		

B)

MS1	Values used
RF Lens	50%
Spray Voltage	1800V
Source Temperature	300℃
Full Scan Range	200-2000 m/z
Automatic Gain Control (AGC) target	Standard
Fill Time Control	Automatic
HCD fragmentation collision energy	28%
Orbitrap resolution (at 200 m/z) for fragmented scans	30,000

C)

MS2	Values used
AGC target	200%
Fill Time Control	Automatic
Isolation Window	1.2m/z
Included Charge States	+2 to +6
Intensity Threshold	5×10^{4}
Dynamic Exclusion	60 secs
Tolerance	±5 ppm

D)

Proteome Discoverer Search Parameters	Values used
Minimum peptide length	6
Maximum peptide length	144
Strict Target FDR	0.01
Relaxed Target FDR	0.05
Maximum Missed Cleavages	2
Precursor Mass Tolerance	10ppm
Fragment Mass Tolerance	0.02 Da
Static Modification	Carbamidomethylation
Dynamic Modifications	Oxidation<br>N-terminal acetylation
